# Nonlinear control of quadrotor UAV under rotor failure for robust trajectory tracking

**DOI:** 10.1038/s41598-025-26264-x

**Published:** 2025-11-26

**Authors:** Ashutosh Simha, Leszek Ambroziak

**Affiliations:** 1 Flight Dynamics and Control Division, Kadet Defense Systems, 89/2 Rampura, Bangalore, 560049 India; 2 Faculty of Mechanical Engineering, Department of Automation Technology, Bialystok University of Technology, Wiejska 45C, 15-351 Białystok, Poland

**Keywords:** Nonlinear control, Geometric control, Reduced attitude tracking, Rotor failure, Quadrotor, Engineering, Aerospace engineering

## Abstract

We present a trajectory tracking control law for a quadrotor unmanned aerial vehicle (UAV) for handling the complete failure of a single rotor. The control design problem considers the reduced state space, which excludes the angular velocity and orientation about the vertical body axis. The proposed controller enables the quadrotor to track the orientation of this axis and consequently any prescribed position trajectory using only three rotors. The control design is carried out in two stages. First, to track the reduced attitude dynamics, a geometric controller with two input torques is designed on the Lie group SO(3). This controller is then extended to SE(3) by designing a saturation-based feedback law to track the center of mass position with bounded thrust. The control law for the complete dynamics achieves an almost global exponential tracking. The novelty of geometric control design lies in its ability to effectively execute aggressive global maneuvers despite the complete loss of a rotor. Numerical simulations on a conventional quadrotor model, carried out for complete trajectory tracking, together with preliminary experimental tests on a real platform under a hover scenario with sudden motor failure, are presented to demonstrate the practical applicability of the proposed control design.

## Introduction

Multirotors are a type of UAV that has been increasingly used in defense, industrial, and civil applications due to their vertical take-off and landing capability, simplified mechanical design (rigidly mounted motors on four arms), and ease of maneuvering (owing to holonomic flight characteristics). The quadrotor consists of two pairs of symmetrically located counter-rotating propeller blades which independently generate aerodynamic thrust along a common axis, in order to regulate the overall applied force and torques on the UAV. In typical flight missions, independent rotor thrusts are regulated in tracking the center-of-mass position and heading angle of the UAV. A basic understanding of the quadrotor dynamics and control design can be found in^1^ and some recent research can be found in^2–6^. In conventional quadrotors, the thrust generated by each rotor is regulated by varying its speed. Such an actuation mechanism has a low control bandwidth due to saturation limits in the electro-mechanical circuit driving the rotor. Furthermore, the rotor thrust must remain strictly positive, which limits the flight envelope. These factors have motivated the development of variable pitch quadrotors^7,8^ in which the rotor thrust is regulated by varying the pitch angle of the propeller blades, while maintaining a constant rotor speed. This mechanism has a significantly higher actuation bandwidth than conventional rotors. Furthermore, the blade pitch angles can be reversed to enable negative thrust generation. It has been shown in^9–11^, that the variable pitch mechanism appreciably enhances the flight envelope, thereby enabling aggressive maneuvers. Notwithstanding these advances in variable-pitch platforms, the predominant operational paradigm – both in practice and in the literature – remains the conventional fixed-pitch quadrotors. A serious limitation of classical multicopters is the failures that occur in the propulsion system. In such a situation, a failure related to the ESC, motor, or propeller causes classic control systems to fail^12^.

The main focus of this paper is to design a control law for a quadrotor after the complete failure of a single rotor. With three functioning rotors, only a three-dimensional submanifold of the output space can be completely regulated. A possible tracking solution is to relinquish control of the angular rate about the thrust axis, and choose the orientation of the thrust axis (i.e. reduced attitude) and net thrust as tracking outputs.

The primary objective of this paper is to develop and validate a nonlinear geometric control strategy for a quadrotor subject to a complete failure of a single rotor. The proposed controller is designed to enable stable flight in the presence of this fault by regulating the reduced attitude and net thrust, while explicitly accounting for the reduced actuation authority. The control law is formulated on the Lie group $\textrm{SE}(3)$ and guarantees exponential tracking performance for admissible initial conditions. To demonstrate the effectiveness of the method, a formal stability analysis is provided, followed by numerical simulations involving both aggressive trajectory tracking and a hover scenario with single-rotor failure, which replicates the conditions of a preliminary experimental test. The proposed approach is particularly suitable for fault-tolerant applications.

### Related works

There is a significant amount of research in fault-tolerant control systems (FTCS) of quadrotors with partial rotor loss. The control systems developed in this group of FTCS include methods such as sliding mode^13–15^), optimal^16^, adaptive^17,18^, or using the active disturbance rejection technique^19^. Reference^20^ proposes a novel approach using the nonlinear model predictive control (NMPC) technique, which leverages the full nonlinear dynamics of the damaged quadrotor’s propulsion system while considering the thrust constraints of each motor-propeller unit. The same group of researchers in the work^21^ describe a novel FTCS control system that combines a fault-tolerant control loop and onboard vision - based state estimation to achieve position control of a quadrotor with complete failure of a single rotor. In this paper, the authors present experimental results that show the effectiveness of the proposed algorithm. An effective reconfiguration mechanism designed to prevent motor failure was presented in^22^. In^23,24^, the authors also presented relaxed hover solutions with multiple rotor failures. The attitude dynamics is linearized about a hovering point where the yaw rate is held at a non-zero constant value. In order to stabilize the position of the UAV, the orientation of the vertical axis (i.e., reduced attitude) and net rotor thrust are regulated. In^25,26^, a PID and a backstepping approach is used for emergency landing in case of rotor failure. In^27,28^, the authors present a hierarchical control design in which the inner loop controls the reduced attitude and the outer loop controls the position. The inner loop consists of a robust feedback linearization-based controller, and the outer loop is an $\textit{H }_{\mathrm {\infty }\ }$- based controller for the translational dynamics, linearized about a hover point. In^29,30^, the authors present linearization-based static and dynamic feedback controllers to regulate the reduced attitude and position of the quadrotor. Control designs based on small-angle or linear approximations restrict the motion of the quadrotor to near-hover maneuvers. Further, feedback linearization-based control laws mentioned above encounter singularities when the roll and pitch angles are $\pi$/2 or when the net thrust is zero. To avoid this, initial state errors must be restricted within a sufficiently small neighborhood of the origin. These factors render the existing fault-tolerant control designs ineffective in tracking global trajectories or performing aggressive maneuvers (such as attitude recovery from an inverted pose). It is imperative to understand that, after rotor failure, the orientation of the quadrotor may undergo large deviations from the operating point, thereby necessitating global maneuvering capability. Recently, Gu and Tian ^31^ proposed a safety control strategy for quadrotors under complete loss of one rotor, which is highly relevant to the scenario considered in this work. Their approach employs a reduced-attitude controller with a disturbance observer and integral action to improve robustness against external disturbances and model uncertainties. They demonstrate, through simulation, that their controller can stabilize the UAV and maintain altitude even after a full rotor failure, by compensating for unmeasured disturbances. In recent years, globally stabilizing geometric controllers that exploit the intrinsic structure of the underlying manifold have gained significant attention. These controllers inherently operate on nonlinear configuration spaces, such as Lie groups, thereby avoiding singularities associated with attitude parameterizations (e.g., Euler angles or quaternions) and circumventing challenges related to input-output decoupling. Foundational contributions, such as those in^32,33^, establish nonlinear proportional-derivative (PD) control laws on Lie groups for general mechanical systems. Building upon these principles,^34,35^ introduced pioneering geometric control frameworks for quadrotor UAVs on the Lie group *SE*(3), enabling almost-global asymptotic stability of both translational and rotational dynamics. More recently, the work by Wu et al.^36^, which presents a geometric maneuvering control strategy for underactuated VTOL vehicles, represents a highly relevant contribution. Their approach offers an innovative outer-loop position control design combined with a geometric attitude control strategy that ensures precise tracking of aggressive maneuvers without requiring full actuation. The reduced stability of the attitude to a fixed point on $\textit{S}{}^{2\ }$with two control torques is presented in^37^. In^38^, the primary axis of a rigid body on $\textit{S}{}^{2\ }$as well as the angular velocity about this axis, are tracked using three independent torques. In^39^, global reduced attitude tracking with three torques is achieved by constructing a synergistic family of potential functions on $\textit{S}{}^{2}$. To the best of our knowledge, none of the above-mentioned control laws are suitable for trajectory tracking with three functioning rotors (i.e. two torque inputs and a net thrust). Fault-tolerant control strategies are not limited to multirotor UAVs but have also been actively explored in the context of fixed-wing platforms. For instance, in^40^, the dynamics of fixed-wing UAVs are systematically decomposed into two subsystems, enabling a dual-loop control approach. The outer loop employs nonlinear model predictive control (NMPC) for accurate trajectory tracking and effective constraint handling, while the inner loop integrates a fault-tolerant sliding mode predictive control (SMPC) to manage actuator faults and disturbances. This combined NMPC–SMPC framework has demonstrated superior performance compared to conventional NMPC trajectory replanning approaches, underscoring the growing relevance of predictive and fault-tolerant methods for UAV applications.

### Contributions

This paper proposes an innovative geometric controller equipped with two input torques, enabling precise tracking of reduced attitude dynamics. Through the implementation of a saturation-based feedback mechanism, the controller gains the capability to maintain the center of mass position while adhering to specified thrust constraints. The comprehensive control law designed for this purpose achieves exponential tracking for all initial conditions within an open and dense subset of the state space. Existing fault-tolerant control strategies for quadrotors experiencing rotor failure primarily rely on linearized dynamics around hover conditions or modified control allocation methods to redistribute thrust among the functional rotors. While these approaches provide basic stabilization, they often encounter significant limitations in aggressive maneuvering and trajectory tracking due to inherent singularities and reduced control authority. In contrast, the proposed nonlinear geometric control framework operates directly on the reduced configuration manifold, eliminating the necessity for local coordinates or linearization and ensuring optimal utilization of the remaining actuation capabilities. Unlike conventional methods, the developed controller facilitates global aggressive maneuvers, such as recovery from inverted poses and robust trajectory tracking, even in the event of complete rotor loss. Furthermore, by designing the control law on the Lie groups SO(3) and SE(3), the approach guarantees singularity-free behavior and exponential convergence properties, representing a substantial advancement over traditional fault-tolerant designs.

## Problem formulation

### Quadrotor dynamics

**Fig. 1:**
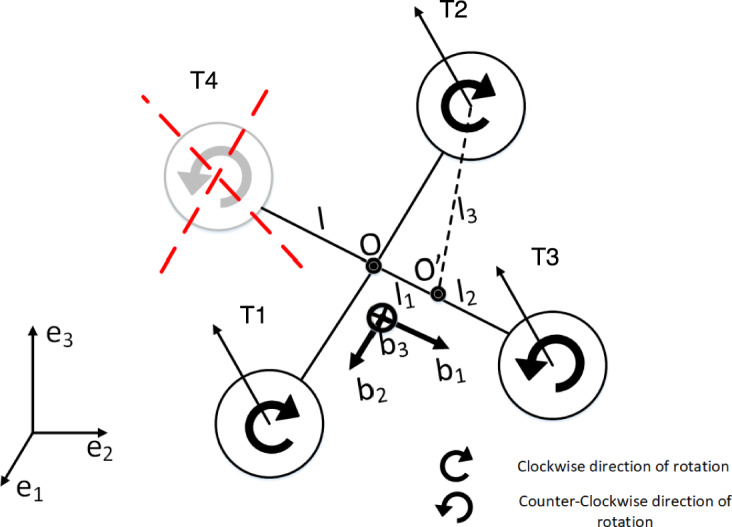
Quadrotor model.

Consider the quadrotor as shown in Fig. 1 . Let $\left\{ \vec {e}_{1}, \vec {e}_{2}, \vec {e}_{3}\right\}$ denote the inertial frame and $\left\{ \vec {b}_{1}, \vec {b}_{2}, \vec {b}_{3}\right\}$ denote the body frame. The four identical rotors are designed to generate thrusts $T_{1}, T_{2}, T_{3}, T_{4}$ along $\vec {b}_{3}$.

In this paper, it is assumed that the fourth rotor has been completely disabled as a result of a fault (i.e. $T_{4} \equiv 0$ ). The origin of the body frame is located at the center of mass. $R \in S O(3)$ is the rotation matrix from the $\vec {b}$ frame to the $\vec {e}$ frame, denoting the attitude of the quadrotor. $\Omega$ is the angular velocity in the body frame. *x* and *v* denote the position and velocity of the center of mass. *m* denotes the mass of the quadrotor, $J=\operatorname {diag}\left( J_{1}, J_{2}, J_{3}\right)$ is the moment of inertia matrix in the body frame, $J_{r}$ is the inertia of the rotors, and $\tau _{d}$ is the aerodynamic rotational drag. The total thrust and torque due to the rotors are represented by *f* and *M* respectively. The first and second rotor spin clockwise and the third spins anti-clockwise at angular speeds $\omega _{1}, \omega _{2}, \omega _{3}$ respectively. The rigid body equations of motion are derived using the Euler-Poincare formalism on *SE*(3) (the configuration manifold of the quadrotor) as follows:

1 $$\begin{aligned} \begin{gathered} \dot{x}=v\, , \\ m \dot{v}=-m g e_{3}+f R e_{3}\, , \\ \dot{R}=R \hat{\Omega }\; , \\ J \dot{\Omega }=J \Omega \times \Omega +g_{r}-\tau _{d}+M , \end{gathered} \end{aligned}$$

where $\hat{\cdot }: \mathbb {R}^{3} \rightarrow \mathfrak {s o}(3)$ is defined as $\hat{x} y=x \times y, x, y \in \mathbb {R}^{3}$. We will denote $(.)^{\vee }$ as its inverse map throughout the paper and $e_{i}$ as the representation of $\vec {e}_{i}$.

When all four rotors are functioning, the gyroscopic moment $g_{r}$ generated by their different rotational speeds is generally neglected. However, $g_{r}$ may be significant in the case of rotor failure and is therefore included. This effect has been modeled as an additional moment by the following equation^41^:

2 $$\begin{aligned} g_{r}=J_{r}\left( \Omega \times e_{3}\right) \left( \omega _{1}-\omega _{2}+\omega _{3}\right) , \end{aligned}$$

where $\omega _{i}$ is the speed of the *i*-th rotor. Aerodynamic drag torque $\tau _{d}$ is difficult to model, as it depends on the quadrotor profile. The model used here has been adopted from^24^, and is based on the form drag of a translating object^42^ which is quadratic in the vehicle’s angular velocity as^5,6^:

3 $$\begin{aligned} \tau _{d}=\Vert \Omega \Vert K_{d} \Omega \; , \end{aligned}$$

where $K_{d}$ is a positive definite matrix.

Let $T_{i}$ and $D_{i}$ denote the thrust and drag induced torque generated by the *i*-th rotor. *f* and *M* are then obtained for the ‘*X*’ configuration of the quadrotor as:

4 $$\begin{aligned} \begin{aligned} f&=T_{1}+T_{2}+T_{3}\, , \\ M_{1}&=d\left( T_{1}-T_{2}-T_{3}\right) , \\ M_{2}&=d\left( T_{1}+T_{2}-T_{3}\right) , \\ M_{3}&=D_{1}-D_{2}+D_{3}\; . \end{aligned} \end{aligned}$$

The thrust and torque are obtained using the following relation ^1^:

5 $$\begin{aligned} T_i&= C_l \omega _r^2 \, , \end{aligned}$$

6 $$\begin{aligned} D_i&= C_d T_i\, , \end{aligned}$$

where $C_l$ and $C_d$ are the aerodynamic lift and drag coefficients, respectively. Key notations and symbols are summarized in Appendix 1.

## Geometric control design

**Fig. 2:**
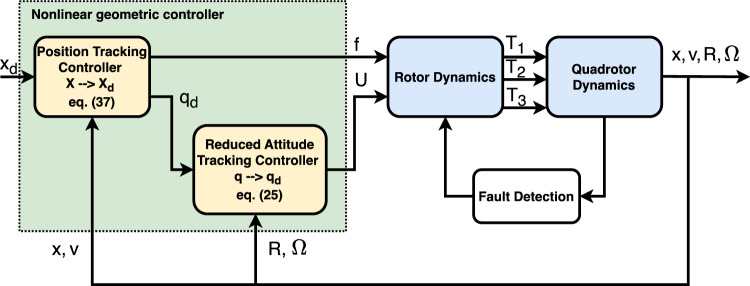
Control system structure.

In this paper, we address the geometric control design of a quadrotor under the condition of a complete failure of one motor. With the three remaining rotors, a control law is designed to globally track the center-of-mass position at an exponential rate. With only three independent controls, it is not reasonable to expect tracking of four independent outputs. For this reason, control of the yaw angle (heading angle) of the quadrotor is relinquished, i.e., tracking of the complete attitude *R* of the quadrotor is not considered. Instead, only its reduced attitude $\textbf{q} = R e_3$, which represents the orientation of the thrust axis and is treated as an input to the translational dynamics, is considered. The proposed control framework explicitly considers two hierarchical levels within the overall control structure (Fig. 2). The first level (inner), the Reduced Attitude Tracking Controller, is responsible for stabilizing the quadrotor’s attitude dynamics on a constrained manifold due to actuator failure. The second level, the Position Tracking Controller, manages the outer-loop translational motion to ensure accurate trajectory tracking despite the underactuated condition. This two-layered control approach enables robust flight performance and precise navigation even in the presence of severe actuator loss. It is assumed that the fault in rotor 4 has already been detected and this rotor has been disabled completely. With three functioning rotors, the control inputs are chosen as *f* and $\textbf{U} = [U_1, U_2]^\top = [M_1/J_1,\, M_2/J_2]^\top$. Control of the angular velocity about the vertical axis, i.e., $\Omega _3$, is relinquished. First, a reduced attitude controller with input $\textbf{U}$ is designed on $\mathbb {S}^2$, which is then extended to a trajectory tracking controller on $\mathbb {S}^2 \times \mathbb {R}^3$ by designing a control law for *f* based on linear-saturation feedback.

### Reduced attitude tracking controller on SO(3)

This section develops a control strategy for the reduced attitude, defined as $q = R e_3 \in \mathbb {S}^2$, to ensure tracking of a twice-differentiable reference trajectory $q_d \in \mathbb {S}^2$. The proposed approach utilizes an output tracking control framework. Here, the reduced attitude represents the direction of the thrust axis (i.e., the body-fixed *z*-axis), which is obtained by projecting the rotation matrix *R* onto the unit sphere $\mathbb {S}^2$ via the mapping $\pi : SO(3) \rightarrow \mathbb {S}^2$, as described in^37^. This projection can be formalized as follows:

7 $$\begin{aligned} \pi (R)=R e_{3}\; . \end{aligned}$$

Let $q=R e_{3}$ denote the reduced attitude, and $\vec {r}_{1}=R e_{1}$, $\vec {r}_{2}=R e_{2}$ denote the two horizontal body axes. The dynamics of *q* can be obtained from (1) as:

8 $$\begin{aligned} \begin{aligned} \dot{q}&=\Omega _{1} \vec {r}_{2}-\Omega _{2} \vec {r}_{1}\; ,\\ \dot{\Omega }_{1}&=\frac{\left( J_{2}-J_{3}\right) }{J_{1}} \Omega _{2} \Omega _{3}+U_{1}+d_{1}(\Omega )\; ,\\ \dot{\Omega }_{2}&=\frac{\left( J_{3}-J_{2}\right) }{J_{2}} \Omega _{1} \Omega _{3}+U_{2}+d_{2}(\Omega )\; . \end{aligned} \end{aligned}$$

Here, $d = [d_1,\; d_2]^\top$ encapsulates the effects of modeling uncertainties associated with the gyroscopic torque and propeller-induced rotational drag. According to their analytical forms presented in (2) and (3), the vector $\textbf{d}$ can be bounded as:

9 $$\begin{aligned} \Vert d\Vert _{2} \le \left( \Vert \tilde{\Omega }\Vert _{2}+\Vert \tilde{\Omega }\Vert _{2}^{2}\right) \Delta \; , \end{aligned}$$

where $$\Delta =\max \left\{ J_{r} \omega _{1}, \lambda _{\max }\left( K_{d}\right) \right\}$$ and $$\tilde{\Omega }=\left[ \Omega _{1}, \Omega _{2}\right] ^{T}$$.

Let $q_{d}(t) \in \mathbb {S}^{2}$ denote the reference reduced attitude trajectory. Motivated by^38,43^, we define a reduced attitude error function as:

10 $$\begin{aligned} \Psi \left( q, q_{d}\right) =2-\frac{2}{\sqrt{2}} \sqrt{1+q_{d}^{T} q}\; . \end{aligned}$$

This choice is motivated by the fact that the Lie derivative of the error function does not vanish when $q \rightarrow -q_{d}$. However, this occurs in the case of the conventional error function on $\mathbb {S}^{2}$ as defined in^33^, thereby rendering the tracking performance poor. It can be observed that the error function satisfies the following:

11 $$\begin{aligned} \begin{gathered} \Psi \left( q, q_{d}\right) \in [0,2], \\ \Psi \left( q, q_{d}\right) =0 \Longleftrightarrow q=q_{d}\; . \end{gathered} \end{aligned}$$

With $q_{d}$ constant, the differential of $\Psi \left( q, q_{d}\right)$ along $T_{q}^{*} \mathbb {S}^{2}$ is computed using the tangent map corresponding to the projection $\pi _{\mathbb {S}^{2}}: \mathbb {R}^{3}-\{\overrightarrow{0}\} \rightarrow \mathbb {S}^{2}$ where $\pi _{\mathbb {S}^{2}}(x)=x /\Vert x\Vert _{2}$, as:

12 $$\begin{aligned} d_{1} \Psi _{\mathbb {S}^{2}}(q, q_d)=\frac{1}{\sqrt{2} \sqrt{1+q_{d}^{T} q}} q \times \left( q \times q_{d}\right) \end{aligned}$$

We denote the reduced attitude error vector as:

13 $$\begin{aligned} e_{q}:=d_{1} \Psi _{\mathbb {S}^{2}}(q, q_d)\; . \end{aligned}$$

Note that this quantity is well defined as long as $q_{d}^{T} q>-1$ i.e. when the angle between them is less than $180^{\circ }$. Before presenting the main stability theorem, we first establish an auxiliary result concerning the relationship between the reduced attitude error function $\Psi$ and the error vector $e_q$. This intermediate step (Lemma 3.1.1) is crucial because it guarantees that the error dynamics remain bounded in a suitable sense. Such a property is necessary to later prove exponential stability of the closed-loop system under rotor failure.

#### Lemma 3.1.1

14 $$\begin{aligned} \left\| e_{q}\right\| _{2}^{2} \le \Psi \le 2\left\| e_{q}\right\| _{2}^{2}, \quad \forall \left( q, q_{d}\right) \in \Psi ^{-1}[0,2), \end{aligned}$$

#### Proof

By using the identity:

$$\begin{aligned} \left\| q_{d}^{T} q\right\| _{2}=\cos (\theta ) \text{ and } \left\| q \times \left( q \times q_{d}\right) \right\| _{2}=\sin (\theta ) \text {, } \end{aligned}$$

where $\theta$ denotes the angle between *q* and $q_{d}$, the inequality (14) follows form (12).

Lemma 3.1.1 shows that the reduced attitude error function $\Psi$ can be bounded above and below by quadratic forms of the error vector $e_q$. This implies that $\Psi$ is locally positive definite with respect to the attitude error and can therefore serve as a component of a Lyapunov candidate function. This property will be exploited in the proof of Theorem 3.1.1.

Next, in order to define the velocity error in $T_{\mathbb {S}^{2}}$, we define the transport map $\mathcal {T}_{\mathbb {S}^{2}}\left( q, q_{d}\right) : T_{q_{d}} \mathbb {S}^{2} \rightarrow T_{q} \mathbb {S}^{2}$^33^ as follows:

15 $$\begin{aligned} \mathcal {T}_{\mathbb {S}^{2}}\left( q, q_{d}\right) \cdot v=\left( q_{d} \times v\right) \times q, \forall v \in T_{q_{d}} \mathbb {S}^{2} \; . \end{aligned}$$

Next, to formalize the connection between the transport map and the error function derivative, we introduce Lemma 3.1.2. This step is needed to compute how the reduced attitude error evolves over time under the proposed control law. We make the following observation:

#### Lemma 3.1.2

The pull-back of the transport map $\mathcal {T}$ satisfies the equation:

16 $$\begin{aligned} \mathcal {T}_{\mathbb {S}^{2}}\left( q, q_{d}\right) ^{*}\left( d_{1} \Psi _{\mathbb {S}^{2}}(q, q d)\right) =-d_{2} \Psi _{\mathbb {S}^{2}}(q, q d)\; . \end{aligned}$$

#### Proof

The equality can be proved (as presented in the chapter 11 in^33^) using the identity:

$$\begin{aligned} (x \times y) \times z=\left( z^{T} x\right) y-\left( z^{T} y\right) x, \forall x, y, z \in \mathbb {R}^{3} . \end{aligned}$$

We now define the velocity error vector as:

17 $$\begin{aligned} e_{\dot{q}}:=\dot{q}-\mathcal {T}_{\mathbb {S}^{2}}\left( q, q_{d}\right) \cdot \dot{q}_{d}\; . \end{aligned}$$

The derivative of the error function can be obtained using Lemma 3.1.2 as:

18 $$\begin{aligned} \begin{aligned} \frac{d}{d t} \Psi \left( q, q_{d}\right)&=d_{1} \Psi _{\mathbb {S}^{2}}\left( q, q_{d}\right) \dot{q}+d_{2} \Psi _{\mathbb {S}^{2}}\left( q, q_{d}\right) \dot{q}_{d}\; , \\&=d_{1} \Psi _{\mathbb {S}^{2}}\left( q, q_{d}\right) e_{\dot{q}}\; . \end{aligned} \end{aligned}$$

In order to stabilize the dynamics of $\Psi$, we require that $e_{\dot{q}}$ satisfies:

19 $$\begin{aligned} e_{\dot{q}}=-k_{q} d_{1} \Psi _{\mathbb {S}^{2}}\; , k_{q}>0\; . \end{aligned}$$

Then, $\dot{\Psi }$ can be obtained as:

20 $$\begin{aligned} \dot{\Psi }=-k_{q}\left\| e_{q}\right\| _{2}^{2}\; . \end{aligned}$$

Further, Lemma 3.1.1 asserts that $\Psi$ can be sandwiched between two positive definite quadratic forms in $e_{q}$, thereby ensuring that the dynamics of $\Psi$ can be bounded as:

21 $$\begin{aligned} \Psi \left( q(t), q_{d}(t)\right) \le \Psi \left( q(0), q_{d}(0)\right) e^{-k_{q} t}\; . \end{aligned}$$

Equation (19) can be written as:

22 $$\begin{aligned} \Omega _{1} \vec {r}_{2}-\Omega _{2} \vec {r}_{1}=\mathcal {T}_{\mathbb {S}^{2}}\left( q, q_{d}\right) \cdot \dot{q}_{d}-k_{q} d_{1} \Psi _{\mathbb {S}^{2}}\; . \end{aligned}$$

Since $\operatorname {span}\left\{ \vec {r}_{1}, \vec {r}_{2}\right\} =T_{q} \mathbb {S}^{2}$, the above equation admits a unique solution for $\Omega _{1}$ and $\Omega _{2}$ that is given by:

23 $$\begin{aligned} \Omega _{d}=\left[ \begin{array}{c} \left\langle \vec {r}_{2},\left( \mathcal {T}_{\mathbb {S}^{2}}\left( q, q_{d}\right) \cdot \dot{q}_{d}-k_{q} d_{1} \Psi _{\mathbb {S}^{2}}\right) \right\rangle \\ \left\langle -\vec {r}_{1},\left( \mathcal {T}_{\mathbb {S}^{2}}\left( q, q_{d}\right) \cdot \dot{q}_{d}-k_{q} d_{1} \Psi _{\mathbb {S}^{2}}\right) \right\rangle \end{array}\right] \; . \end{aligned}$$

We now construct a control law to track a commanded reduced attitude trajectory. Define:

$$\begin{aligned} e_{\Omega }&:=\left( \left[ \Omega _{1}, \Omega _{2}\right] ^{T}-\Omega _{d}\right) \; , \\ f_{J}(\Omega )&:=\left[ \frac{\left( J_{2}-J_{3}\right) }{J_{1}} \Omega _{2} \Omega _{3}, \frac{\left( J_{3}-J_{1}\right) }{J_{2}} \Omega _{1} \Omega _{3}\right] ^{T}\; , \end{aligned}$$

and

24 $$\begin{aligned} U_{\Delta }=\left\{ \begin{array}{ll} \frac{e_{\Omega }}{\left\| e_{\Omega }\right\| _{2}}, & \left\| e_{\Omega }\right\| _{2}> \text{ tol } \\ \frac{e_{\Omega }}{ \text{ tol } }, & \left\| e_{\Omega }\right\| _{2} \le \text{ tol } \end{array}\right\} \; , \end{aligned}$$

where tol $>0$ is a positive constant depending on the slew rate of the torque actuation.

#### Theorem 3.1.1

Given a reference trajectory $q_{d}(t)$ that is smooth with bounded derivatives, the control law:

25 $$\begin{aligned} \begin{aligned} U(R, \Omega , t) :=\quad&-\alpha \left[ \begin{array}{c} \left\langle d_{1} \Psi _{\mathbb {S}^{2}}, \vec {r}_{2}\right\rangle \\ \left\langle d_{1} \Psi _{\mathbb {S}^{2}}, -\vec {r}_{1}\right\rangle \end{array}\right] - k_{\Omega } e_{\Omega } - f_{J}(\Omega ) \\&+ \dot{\Omega }_{d} - U_{\Delta }\left( \Vert \tilde{\Omega }\Vert _{2}+\Vert \tilde{\Omega }\Vert _{2}^{2}\right) \Delta \; . \end{aligned} \end{aligned}$$

ensures that $e_{q}$ and $e_{\Omega }$ exponentially converge to an arbitrarily small open neighborhood of the origin, for all initial conditions in the open-dense sublevel set $\Psi ^{-1}[0,2)$ satisfying:

26 $$\begin{aligned} \left\| e_{\Omega }(0)\right\| _{2}<2 \alpha (2-\Psi (0))\; . \end{aligned}$$

Further, the sublevel set $\Psi ^{-1}[0,2)$ remains invariant for the closed-loop flow of (8) with the feedback law (25).

#### Proof

Consider the Lyapunov function:

27 $$\begin{aligned} V_{1}:=\alpha \Psi +\frac{1}{2}\left\| e_{\Omega }\right\| _{2}^{2}\; . \end{aligned}$$

Its derivative along the trajectories of 8 with the control law (25), is obtained using (18), (23), (24), and (25), as follows after appropriate substitutions:

28 $$\begin{aligned} \begin{aligned} \dot{V}_{1}=&-\alpha k_{q}\left\| e_{q}\right\| _{2}^{2}-k_{\Omega }\left\| e_{\Omega }\right\| _{2}^{2} \\&+\left\langle e_{\Omega }, d-U_{\Delta }\left( \Vert \tilde{\Omega }\Vert _{2}+\Vert \tilde{\Omega }\Vert _{2}^{2}\right) \Delta \right\rangle \; . \end{aligned} \end{aligned}$$

When $\left\| e_{\Omega }\right\| _{2}>$ tol, the inner product term can be shown to be negative using (9) along with the Cauchy-Schwarz inequality. When $\left\| e_{\Omega }\right\| _{2} \le$ tol, a straightforward calculation shows that we can bound the inner product term using (9), as:

29 $$\begin{aligned} \left\langle e_{\Omega }, d-U_{\Delta }\left( \Vert \tilde{\Omega }\Vert _{2}+\Vert \tilde{\Omega }\Vert _{2}^{2}\right) \Delta \right\rangle \le \operatorname {tol} \Delta \left( \Vert \tilde{\Omega }\Vert _{2}+\Vert \tilde{\Omega }\Vert _{2}^{2}\right) \; . \end{aligned}$$

Further, since $\dot{q}_{d}$ is bounded, $\Omega _{d}$ can be uniformly bounded.

When $\left\| e_{\Omega }\right\| _{2}<$ tol, one can show that $\Vert \tilde{\Omega }\Vert _{2}$ is uniformly bounded within a neighborhood of $\Omega _{d}$, using the triangle inequality. Therefore, by appropriately selecting the value of tol, the inner product term can be bounded above by an arbitrarily chosen constant $\epsilon >0$ as:

30 $$\begin{aligned} \left\langle e_{\Omega }, d-U_{\Delta }\left( \Vert \tilde{\Omega }\Vert _{2}+\Vert \tilde{\Omega }\Vert _{2}^{2}\right) \Delta \right\rangle \le \epsilon \; . \end{aligned}$$

With this, the derivative of $V_{1}$ can be bounded as:

31 $$\begin{aligned} \dot{V}_{1} \le -\alpha k_{q}\left\| e_{q}\right\| _{2}^{2}-k_{\Omega }\left\| e_{\Omega }\right\| _{2}^{2}+\epsilon \; . \end{aligned}$$

From 3.1.1 we have,

32 $$\begin{aligned} -\alpha k_{q}\left\| e_{q}\right\| _{2}^{2} \le -\frac{\alpha k_{q}}{2} \Psi \; , \end{aligned}$$

therefore,

33 $$\begin{aligned} \dot{V}_{1} \le -\beta V_{1}+\epsilon \; , \end{aligned}$$

where $\beta =\min \left( \frac{k_{q}}{2}, k_{\Omega }\right)$. Further, since $\epsilon$ can be arbitrarily defined, $V_{1}$ can be guaranteed to be strictly monotonically decreasing in $\Psi ^{-1}(\bar{\epsilon }, 2)$ where $\bar{\epsilon }$ can be made arbitrarily small. In this region,

34 $$\begin{aligned} \alpha \Psi (t) \le V_{1}(0) \le \alpha \Psi (0)+\frac{1}{2}\left\| e_{\Omega }(0)\right\| _{2}^{2}\; . \end{aligned}$$

Therefore, applying the condition (26), we obtain:

35 $$\begin{aligned} \alpha \Psi (t) \le 2 \alpha \; , \forall \Psi (0) \in (\bar{\epsilon }, 2)\; . \end{aligned}$$

Since $\bar{\epsilon }$ was arbitrary, the sublevel set $\Psi ^{-1}[0,2)$ remains invariant.

#### Remark 3.1

In this control approach, the main design parameters are $k_{q}$, $k_{\Omega }$, $\alpha$, and $\Delta$. Selecting a larger value for $\alpha$ guarantees that the controller remains continuous even at $\Psi = 2$. This high gain is particularly important in cases where the initial angular velocity error is significant. The parameters $k_{q}$ and $k_{\Omega }$ can be adjusted freely to set the desired exponential convergence rate. Lastly, increasing $\Delta$ serves to reduce the upper bound on the steady-state tracking error.

#### Remark 3.2

If employing a high gain $\alpha$ is not feasible, an alternative is to smooth the error function $\Psi$ so that $d_{1}\Psi$ remains continuous at $\Psi =2$. One such smoothing is to use the classical error function, namely $\Psi = 1 - q_{d}^T q$. With the control law as in Eq. (24), the time derivative of the Lyapunov candidate $V_1$ is given by Eq. (30). However, under this modification, it is possible for $\dot{V}_1$ to become zero when $\Psi =2$ and $e_{\Omega }=0$. In this scenario, the LaSalle-Yoshizawa theorem^44^ can be invoked to establish that the trajectories ultimately converge to the set defined by $e_q=0$ and $e_{\Omega }=0$. Note that $e_q=0$ occurs for both $q=q_d$ and $q=-q_d$. Therefore, it becomes necessary to demonstrate that the undesired equilibrium at $q=-q_d$ is locally unstable (at least when $\epsilon \approx 0$).

Consider a function $W=2 \alpha -V_{1}$ that vanishes when $q=-q_{d}$. From the continuity of $\Psi$, it can be shown that in any arbitrarily small neighborhood of $\left( q, e_{\Omega }\right) =\left( -q_{d}, 0\right)$, there exist points *q* where $2-\Psi >0$. At such points, when $e_{\Omega }$ is small enough, it can be shown that $W>0$. Furthermore, in an open neighborhood of the undesired equilibrium point (excluding it), $\dot{W}=-\dot{V}>0$. Since the complement set of the equilibria is positively invariant, Chetaev’s theorem^45^ can be applied to conclude that the undesired equilibria are unstable. Hence, the trajectories of the system converge asymptotically to the stable equilibrium $\left( \Psi , e_{\Omega }\right) =(0,0)$, for almost all initial conditions.

Note, however, that such analysis may not be valid when $\epsilon$ is significant, thereby further justifying our choice of error function.

### Position tracking controller on *SE*(3)

Let $x_{d}$ denote a smooth reference trajectory for the position of the center of mass. We assume that $x_{d}$ and its derivatives are bounded. Let the position and velocity errors be defined as

$$e_{x} := x - x_{d}\; ,$$

$$e_{v} := \dot{x} - \dot{x}_{d}\; .$$

A saturation-based feedback law is now designed to track the position trajectory with bounded thrust.

#### Definition 3.2.1

Given constants *a* and *b* such that $0<a \le b$, a function $\sigma : \mathbb {R} \rightarrow \mathbb {R}$ is said to be a smooth linear saturation function with limits (*a*, *b*), if it is smooth and satisfies:

1. $$s \sigma (s)>0, \forall s \ne 0$$ ,
2. $$\sigma (s)=s, \forall |s| \le a$$ ,
3. $$|\sigma (s)| \le b, \forall s \in \mathbb {R}$$ .

It is well known that such smooth saturation functions exist. For example, consider the integral of a smooth function with compact support, which is constant within a subinterval of its support^46^. Such a function, when shifted by a constant, satisfies the conditions in the definition. In practice, one can approximate these functions using polynomials.

Let $\sigma _{1}$ and $\sigma _{2}$ be two saturation functions with limits $\left( a_{1}, b_{1}\right)$ and $\left( a_{2}, b_{2}\right)$ such that,

36 $$\begin{aligned} b_{1}<\frac{a_{2}}{2}\; . \end{aligned}$$

We now define a control law for $\hat{f}$ which is the total vector thrust acting on the rigid body, as follows:

37 $$\begin{aligned} \hat{f}=\bar{\sigma }\left( e_{x}, e_{v}\right) +f_{d}\; , \end{aligned}$$

where

38 $$\begin{aligned} f_{d}= & m \ddot{x}_{d}+m g e_{3}\;, \end{aligned}$$

39 $$\begin{aligned} \bar{\sigma }\left( e_{x}, e_{v}\right)= & -\left[ \begin{array}{c} \sigma _{2}\left( \frac{k_{1}}{k_{2}} e_{v_{1}}+\sigma _{1}\left( k_{2} m e_{v_{1}}+k_{1} e_{x_{1}}\right) \right. \\ \sigma _{2}\left( \frac{k_{1}}{k_{2}} e_{v_{2}}+\sigma _{1}\left( k_{2} m e_{v_{2}}+k_{1} e_{x_{2}}\right) \right. \\ \sigma _{2}\left( \frac{k_{1}}{k_{2}} e_{v_{3}}+\sigma _{1}\left( k_{2} m e_{v_{3}}+k_{1} e_{x_{3}}\right) \right. \end{array}\right] \;, \end{aligned}$$

and $k_{1}, k_{2}$ are positive constants.

When $f R e_{3}=\hat{f}$, it can be established from Theorem 2.1 in^47^ that the tracking errors enter the linear region of the saturation functions in finite time, and remain within thereafter. This would ensure that the origin of the tracking errors is exponentially attractive. We refer the reader to^48^ for a proof of stability of position controller.

The following Lemma will be subsequently used to demonstrate that the tracking errors enter the linear region in finite time, when the reduced attitude error is sufficiently bounded. To establish convergence of the translational error dynamics, we first show in Lemma 3.2.1 that the tracking errors enter the linear region of the saturation functions in finite time. This property is necessary to guarantee exponential convergence in Theorem 3.2.1

#### Lemma 3.2.1

Let $\sigma _1$ and $\sigma _2$ be saturation functions with limits as prescribed in (36). Then, the trajectories of the system:

$$\begin{aligned} \dot{y}_1&=y_2\; , \\ m \dot{y}_2&=-\sigma _2\left( \left( k_1 / k_2\right) y_2+\sigma _1\left( k_1 y_1+k_2 m y_2\right) \right) +\xi (t)\; , \end{aligned}$$

enter the linear region of $\sigma _1$ and $\sigma _2$ in a finite time $t_2$ and remain within thereafter if,

$$\begin{aligned} |\xi (t)|<\min \left( \left( a_2 / 2\right) -b_1, a_1\right) \; , \forall t>0\; . \end{aligned}$$

#### Proof

Let $w_1=m y_2^2$. We obtain,

40 $$\begin{aligned} \dot{w}_1=2 y_2\left( -\sigma _2\left( \left( k_1 / k_2\right) y_2+\sigma _1\left( k_1 y_1+k_2 m y_2\right) \right) +\xi (t)\right) \; . \end{aligned}$$

When $\left| y_2\right| \ge \left( k_2 / k_1\right) \left( a_2 / 2\right)$, using the bound on $\xi$ and (36), we can see that $\dot{w}_1$ is uniformly negative definite.

Hence, $\exists t_1>0, \quad \ni \left| y_2(t)\right| <\left( k_2 / k_1\right) \left( a_2 / 2\right) , \quad \forall t>t_1$. Using the bound on $b_1$, we conclude that $\sigma _2$ operates in its linear region after $t_1$.

Let $w_2=\left\| \left( k_1 y_1+k_2 m y_2\right) \right\| _2^2$. When $t>t_1$, its derivative is obtained as,

41 $$\begin{aligned} \dot{w}_2=-2\left( k_1 y_1+k_2 m y_2\right) \left( \sigma _1\left( k_1 y_1+k_2 m y_2\right) +\xi (t)\right) \; . \end{aligned}$$

From the definition of $\sigma _1$ and the bound on $\xi , w_2$ is uniformly negative definite when $\left| k_1 y_1+k_2 m y_2\right| \ge a_1$.

Hence, $\exists t_2>t_1>0, \quad \ni \left| k_1 y_1+k_2 m y_2\right| <a_1, \forall t>t_2$. It can there be concluded that $\sigma _1$ and $\sigma _2$ operate in their respective linear regions after $t_2$.

Thus, Lemma 3.2.1 confirms that the saturation-based feedback law drives the tracking errors into the linear region, enabling the subsequent Lyapunov analysis of the translational dynamics. Once the error enters the linear region in finite time, the convergence is exponential thereafter. We now define the commanded reduced attitude trajectory as:

42 $$\begin{aligned} q_d=\frac{\hat{f}}{\Vert \hat{f}\Vert _2}\; . \end{aligned}$$

This is well defined when $\Vert \hat{f}\Vert _2$ is bounded away from zero. One way to ensure this is to choose a bound on $b_2$ as:

$$\begin{aligned} b_2<\inf _{t>0}\left\{ \left\| f_d(t)\right\| _{\infty }\right\} \; . \end{aligned}$$

The control law for the net thrust *f* is then chosen as:

43 $$\begin{aligned} f=\left\langle \hat{f}, R e_3\right\rangle \; . \end{aligned}$$

Having established the necessary intermediate results (Lemmas 3.1.1, 3.1.2, and 3.2.1), we are now in a position to prove the main stability result for the full closed-loop system.

#### Theorem 3.2.1

Consider the control law for *U* and *f* as given in (25) and (43) such that the condition (26) is satisfied. Further, define the matrices:

44 $$\begin{aligned} \begin{aligned}&W_1=\left[ \begin{array}{cc} \frac{c k_x}{m}\left( 1-\sin \left( \theta _0\right) \right) & -\frac{c k_v}{2 m}\left( 1+\sin \left( \theta _0\right) \right) \\ -\frac{c k_v}{2 m}\left( 1+\sin \left( \theta _0\right) \right) & k_v\left( 1-\sin \left( \theta _0\right) \right) -c \end{array}\right] , \\&W_2=2\left[ \begin{array}{cc} (c / m)\left\| f_d\right\| _2 & 0 \\ a_1+\left\| f_d\right\| _2 & 0 \end{array}\right] . \end{aligned} \end{aligned}$$

Given $0<k_x:=k_1, 0<k_v:=\left( k_1 / k_2\right) +k_2$, and $\theta _0<$ $\pi / 2$, we choose positive constants $c, k_q, k_{\Omega }$, such that:

45 $$\begin{aligned} \begin{aligned}&c<\min \left\{ k _ { x } k _ { v } ( 1 - \operatorname { s i n } ( \theta _ { 0 } ) ) ^ { 2 } \left( k_x\left( 1-\sin \left( \theta _0\right) \right) \right. \right. \\&\left. \left. +\frac{k_v^2\left( 1+\sin \left( \theta _0\right) \right) ^2}{4 m}\right) ^{-1}, k_v\left( 1-\sin \left( \theta _0\right) \right) , \sqrt{k_x / m}\right\} , \\&\min \left( \alpha k_q, k_{\Omega }\right) >\frac{4\left\| W_2\right\| ^2}{\lambda _{\min }\left( W_1\right) }\; . \end{aligned} \end{aligned}$$

Then, the tracking errors $e_x, e_v, e_q, e_{\Omega }$, exponentially converge to an arbitrarily small open neighborhood of the origin, for all initial conditions lying in an open-dense subset.

#### Proof

Assuming that $\theta <\frac{\pi }{2}$, the dynamics of $e_v$ can be written using (1) as:

46 $$\begin{aligned} m \dot{e}_v=-m g e_3-m \ddot{x}_d+\frac{f}{q_d^T q} q_d+\mathcal {E}\; , \end{aligned}$$

where $\mathcal {E} \in \mathbb {R}^3$ is defined as:

47 $$\begin{aligned} \mathcal {E}=f\left( q-\frac{q_d}{q_d^T q}\right) \; . \end{aligned}$$

Further, we can write that:

48 $$\begin{aligned} \frac{f}{q_d^T q} q_d=\frac{\Vert \hat{f}\Vert _2 q_d^T q}{q_d^T q} \cdot \frac{\hat{f}}{\Vert \hat{f}\Vert _2}=\hat{f}\; . \end{aligned}$$

Hence, using (37) we can write:

49 $$\begin{aligned} m \dot{e}_v=\bar{\sigma }\left( e_x, e_v\right) +\mathcal {E}\; . \end{aligned}$$

From (47), we can bound $\mathcal {E}$ as:

50 $$\begin{aligned} \mathcal {E} \le \Vert \hat{f}\Vert _2\left\| \left( \left( q_d^T q\right) q-q_d\right) \right\| _2\; , \end{aligned}$$

where the term $\left\| \left( \left( q_d^T q\right) q-q_d\right) \right\| _2=|\sin (\theta )|$. Further, the commanded thrust $\hat{f}$ can be bounded using the saturation limit $b_2$ as:

51 $$\begin{aligned} \Vert \hat{f}\Vert _2 \le \sqrt{3} b_2+\sup _{t>0}\left\{ \left\| f_d(t)\right\| _2\right\} :=B\; . \end{aligned}$$

From Theorem 3.1.1 we know that,

52 $$\begin{aligned} \exists t_0>0, \quad \ni |\sin (\theta )|<\min \left( \frac{\delta }{B},\left| \sin \left( \theta _0\right) \right| \right) , \forall t>t_0\; , \end{aligned}$$

This implies that $\Vert \xi (t)\Vert _{\infty }<\delta , \quad \forall t>t_0$, where $\delta :=\min \left( \left( a_2 / 2\right) -b_1, a_1\right)$.

Using Lemma 3.2.1, we can then conclude that the error dynamics $e_x$ and $e_v$ operate in the linear region of $\sigma _1$ and $\sigma _2$ after a finite time $t_0+t_2$. Further, if $\Vert \hat{f}\Vert _2$ is bounded away from zero, the reference trajectory $q_d$ is well defined and its derivatives are bounded. Therefore, the trajectories of (1) remain bounded in $\left( 0, t_0+t_2\right)$.

In the linear region, the dynamics of $e_v$ can be written as:

53 $$\begin{aligned} m \dot{e}_v=-k_x e_x-k_v e_v+\mathcal {E}\; . \end{aligned}$$

We choose a Lyapunov function candidate for the translational dynamics as:

54 $$\begin{aligned} V_2:=\frac{1}{2} k_x\left\| e_x\right\| _2^2+\frac{1}{2} m\left\| e_v\right\| _2^2+c e_x^T e_v\; . \end{aligned}$$

Its derivative along the flow of (53) is obtained as:

55 $$\begin{aligned} \dot{V}_2=-\left( k_v-c\right) \left\| e_v\right\| _2^2-\frac{c k_x}{m}\left\| e_x\right\| _2^2-\frac{c k_v}{m}+\mathcal {E}^T\left( \frac{c}{m} e_x+e_v\right) \; . \end{aligned}$$

From (52) we observe that:

56 $$\begin{aligned} \left\| \left( \left( q_d^T q\right) q-q_d\right) \right\| _2<1, \forall t>t_0\; . \end{aligned}$$

Further, from the identity given in the proof of Lemma 3.1.1, we observe that:

57 $$\begin{aligned} \left\| \left( \left( q_d^T q\right) q-q_d\right) \right\| _2 \le 2\left\| e_q\right\| _2\; . \end{aligned}$$

This can be substituted in (55) to obtain:

58 $$\begin{aligned} \begin{array}{r} \dot{V}_2 \le -\left( k_v(1-\sin (\theta ))-c\right) \left\| e_v\right\| _2^2-\frac{c k_x}{m}(1-\sin (\theta ))\left\| e_x\right\| _2^2 \\ +\frac{c k_v}{m}(1+\sin (\theta ))\left\| e_x\right\| _2\left\| e_v\right\| _2 \\ +2\left\| e_q\right\| _2\left( k_x\left\| e_x\right\| _2\left\| e_v\right\| _2+\frac{c}{m}\left\| f_d\right\| _2\left\| e_x\right\| _2+\left\| f_d\right\| _2\left\| e_v\right\| _2\right) \; . \end{array} \end{aligned}$$

In the linear region of the saturation functions, we can bound the cubic term in the above equation as:

59 $$\begin{aligned} \left\| e_q\right\| _2 k_x\left\| e_x\right\| _2\left\| e_v\right\| _2 \le a_1\left\| e_q\right\| _2\left\| e_v\right\| _2\; . \end{aligned}$$

Consider a Lyapunov function candidate for the complete dynamics as:

60 $$\begin{aligned} V=V_1+V_2\; , \end{aligned}$$

where $V_1$ is defined as in (27).

Define: $z_1=\left[ \left\| e_x\right\| _2,\left\| e_v\right\| _2\right] ^T$ and $z_2=\left[ \left\| e_q\right\| _2,\left\| e_{\Omega }\right\| _2\right] ^T$. We can bound *V* between two quadratic forms using Lemma 3.1.1 as:

61 $$\begin{aligned} z_1^T M_1 z_1+z_2^T M_2 z_2 \le V \le z_1^T M_3 z_1+z_2^T M_4 z_2\; , \end{aligned}$$

where

62 $$\begin{aligned} \begin{gathered} M_1=\frac{1}{2}\left[ \begin{array}{cc} k_x & -c \\ -c & m \end{array}\right] \; , \quad M_3=\frac{1}{2}\left[ \begin{array}{cc} k_x & c \\ c & m \end{array}\right] \; , \\ M_2=\frac{1}{2}\left[ \begin{array}{cc} 2 \alpha & 0 \\ 0 & 1 \end{array}\right] \; , \quad M_4=\frac{1}{2}\left[ \begin{array}{cc} 4 \alpha & 0 \\ 0 & 1 \end{array}\right] \; . \end{gathered} \end{aligned}$$

From (31), (58), (59) and (60), the derivative of *V* can be obtained as:

63 $$\begin{aligned} \dot{V} \le -Q\left( e_x, e_v, e_q, e_{\Omega }\right) +\epsilon \; , \end{aligned}$$

where:

64 $$\begin{aligned} Q\left( e_x, e_v, e_q, e_{\Omega }\right) =z_1^T W_1 z_1-z_1^T W_2 z_2+z_2^T W_3 z_2\; , \end{aligned}$$

and

$$W_3=\left[ \begin{array}{cc}\alpha k_q & 0 \\ 0 & k_{\Omega }\end{array}\right] , W_1$$ and $W_2$ are as given in (44).

Using the conditions in (45), we observe that *Q* is a positive definite quadratic form and *V* is sandwiched between two positive definite quadratic forms. Hence, after a finite time $t_0+t_2$, the errors $\left[ e_x, e_v, e_q, e_{\Omega }\right]$ exponentially converge to an arbitrarily small open neighborhood of the origin.

In Section “Position tracking controller on *SE*(3)”, we extend the analysis to the translational subsystem. The key difficulty here arises from the underactuation introduced by the rotor failure, which reduces the available control authority. To address this, Lemma 3.1.2 establishes that the tracking errors enter the linear region of the saturation functions, which allows us to apply Lyapunov analysis in a standard quadratic form. This step is essential for proving exponential convergence in Theorem 3.2.1.

#### Remark 3.3

By employing saturated thrust feedback, the translational error $\mathcal {E}$ can be effectively bounded by sufficiently reducing the initial reduced attitude error during the phase $t < t_0$. This approach is crucial to guarantee that the position errors decrease to the linear region within a finite time interval. Consequently, it becomes possible to bound the cubic term as shown in (59), which establishes exponential stability. In^18^, the stability analysis for translational dynamics is limited to regions where $e_x$ remains bounded. Achieving this requires additional constraints on the total system errors, thereby restricting overall stability to be only local. Similarly, in^34^, the authors focus on bounding the velocity error $e_v$, but their analysis holds only if the gain $k_x$ is identically zero; otherwise, no uniform bound on $e_v$ can be obtained. In contrast, the stability analysis for the proposed control strategy in this work is not subject to these limitations.

#### Remark 3.4

The theorem requires that the attitude tracking gains be sufficiently large to drive the translational errors into the linear regime of the saturation function. The saturation limits selected in this work are intentionally conservative, providing a margin that keeps the commanded thrust vector *f* away from the origin. As a result, both $\dot{q}_d$ and $\ddot{q}_d$ remain bounded, which in turn constrains the control torque. Nonetheless, in practical scenarios, these limits can be relaxed further, as long as the resulting thrust remains within the actuator (rotor) saturation bounds.

In summary, the results of Sections “Reduced attitude tracking controller on SO(3)” and “Position tracking controller on *SE*(3)” establish that the proposed geometric control law guarantees exponential tracking of the reduced attitude and bounded tracking of the translational dynamics under complete failure of a single rotor. These theoretical guarantees provide the foundation for the numerical and experimental validation presented in the Section “Numerical simulations”.

### Implementation note

To facilitate practical implementation and enhance the clarity of our approach, we summarize below the main steps of the proposed control algorithm in the form of a pseudocode (Algorithm 1). This explicit formulation highlights the key computations required in each control cycle and is intended to guide readers interested in real-world deployment of the presented geometric control law. The algorithm reflects the hierarchical structure of the controller, in which the outer loop determines the required thrust and reference direction based on position tracking objectives, while the inner loop computes the necessary attitude torques to align the actual thrust direction with the reference. All feedback signals, including position, velocity, orientation, and angular velocity, are assumed to be available through state estimation or onboard sensors, as is standard in practical UAV systems. Furthermore, the presented procedure specifies how the control inputs (total thrust and reduced attitude torques) should be generated and provided to the quadrotor dynamics, accounting for the actuator limitations and loss of yaw authority that arise under single rotor failure. By explicitly stating each computational step, this note aims to bridge the gap between theoretical development and practical implementation.

**Algorithm 1:**
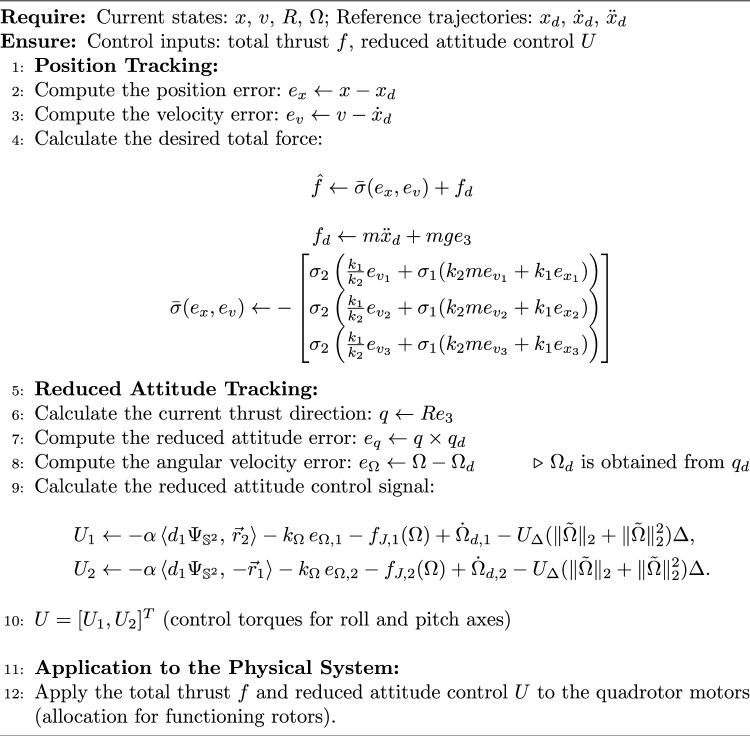
Implementation of the Geometric Control Law for Quadrotor Under Rotor Failure

## Numerical simulations

**Fig. 3:**
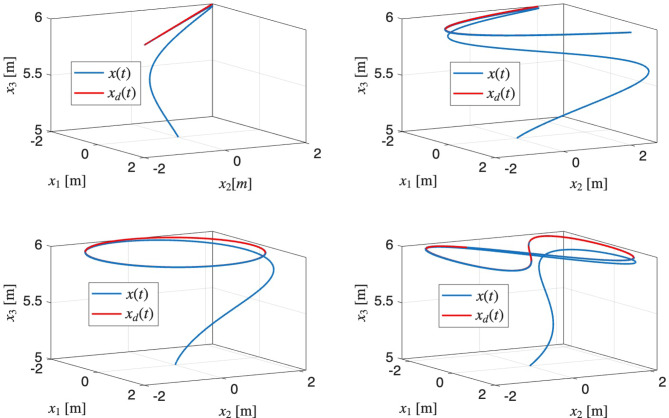
Examples of tested trajectory shapes.

We report results along two complementary tracks: (i) aggressive maneuver simulations (with and without external disturbances) that stress test the controller in idealized conditions and demonstrate its full capability (e.g., large attitude excursions, inverted recovery) and (ii) hover scenario with sudden motor fault injection that is used both in simulation and on the physical platform to directly validate the controller in realistic lab conditions. The former probes the theoretical limits of performance; the latter provides a like-for-like comparison with experimental data. Simulations were carried out on a conventional quadrotor with a positive rotor thrust constraint. Subsequent to rotor failure, the quadrotor was required to track a few trajectory shapes (straight line, curve line, circle, eight) defined with Lissajous curves while initially recovering from a downward facing pose. The Lissajous curves allow one to test the tracking quality with various shapes of trajectories (straight line, arc, circle, eight shape, and other more advanced ones) by changing only 3 parameters of the curve (Fig. 3). The article presents the results for the case of eight shape trajectory tracking without and with disturbances, because this shape of the curve includes features of circle tracking and changes in the quadrotor configuration. Simulations were carried out for ideal conditions with and without the disturbances applied to the quadrotor position, which can imitate wind gusts. The quadrotor parameters chosen for the simulations are $m=1 \mathrm {~kg}, \omega _{i}=600 \textrm{rad} / \textrm{s}$ and $b_{L}=3.2 \times 10^{-6}$, and the inertia matrix is as follows:

$$J=\left[ \begin{array}{lll} 0.0972 & 0.0194 & 0.0195 \\ 0.0194 & 0.0974 & 0.0317 \\ 0.0195 & 0.0317 & 0.1584 \end{array}\right] \; .$$

The nominal inertial matrix for control design was chosen as: $J_{0}=\operatorname {diag}(0.081,0.0812,0.1320)$. The inertia of the propeller was chosen as $J_{r}=5 \times 10^{-5}$, and the rotational drag coefficient matrix was chosen as $K_{d}=\operatorname {diag}(0.7,0.7,1.4) \times 10^{-4}$.

The control gains were chosen as $k_{q}=8, k_{\Omega }=10, k_{x}=$ $2, k_{v}=3, \Delta =3 \times 10^{-3}$, tol $=10^{-3}$. The gains were tuned on the basis of a simple step-response analysis, without the use of advanced optimization methods. The tuning process is straightforward and comparable in complexity to that of a classical PID controller. In future work, we intend to explore automated tuning techniques, such as genetic algorithms, to further refine gain selection in real time.

The initial conditions were chosen as $x(0)=[0,0,5]$, $\dot{x}(0)=0, \Omega (0)=0$, and an initial orientation as a $140^{\circ }$ rotation about the *x* axis as:

$$R(0)=\left[ \begin{array}{ccc} 1 & 0 & 0 \\ 0 & \cos \left( 140^{\circ }\right) & -\sin \left( 140^{\circ }\right) \\ 0 & \sin \left( 140^{\circ }\right) & \cos \left( 140^{\circ }\right) \end{array}\right] \; .$$

To assess the robustness of the proposed control system to parameter variations, a Monte Carlo analysis was performed with respect to the quadrotor parameters eg. mass. The nominal mass was set to $m = 1$ kg, and its value was randomly sampled from a uniform distribution within $\pm 50\%$ of the nominal value, resulting in $N_{\text {trials}} = 30$ simulation runs with different mass realizations. For each trial, the closed-loop tracking error was recorded over time for all position axes (*x*, *y*, *z*).

To visualize the variability of the tracking performance under mass uncertainty, an envelope plot was generated for each axis. The plot shows the median and 5th–95th percentile range of the absolute position tracking errors across all trials at each time instant, as well as individual sample trajectories. This approach provides a comprehensive view of both typical and worst-case controller performance under significant parametric uncertainty. The results demonstrate the ability of the proposed control method to maintain satisfactory tracking accuracy, despite large deviations in system mass.

### Trajectory tracking without external disturbances

**Fig. 4:**
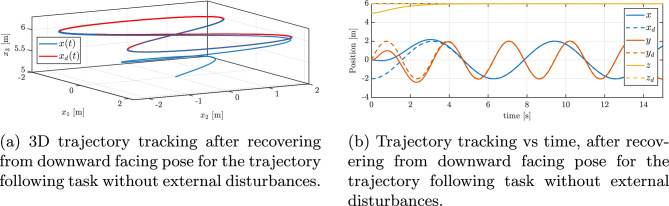
Trajectory tracking performance after recovering from downward facing pose: (**a**) 3D trajectory, (**b**) position vs. time.

**Fig. 5:**
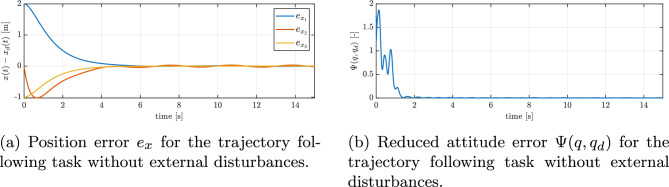
Trajectory tracking errors without external disturbances: (**a**) position error, (**b**) reduced attitude error.

Figure 4a and b shows the quadrotor tracking a ‘figure-of-8’ reference trajectory after recovering from an inverted pose. Initially, when the reduced attitude error is large, there is a transient deviation from the reference trajectory. This can be seen in the position error plot in Fig. 5a. From this plot, it can also be seen that the tracking errors exponentially decrease and are bounded within an arbitrarily small open ball.

**Fig. 6:**
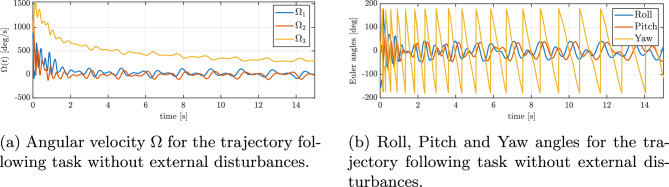
Attitude dynamics during trajectory following without external disturbances: (**a**) angular velocities, (**b**) Euler angles.

**Fig. 7:**
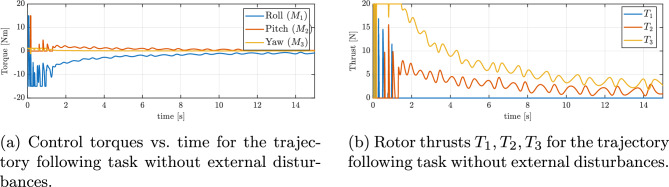
Control inputs during trajectory following without external disturbances: (**a**) commanded torques, (**b**) rotor thrusts.

Figure 5b shows the evolution of the reduced attitude error function $\Psi \left( q, q_{d}\right)$ during the maneuver. It can be seen that $\Psi$ decreases exponentially to an arbitrarily small open ball. Figure 6a shows the angular velocity about the three body axes during the maneuver. It can be seen that the angular velocity about the body *z* axis increases rapidly and saturates due to rotational drag. Figure 6b presents the evolution of roll, pitch, and yaw (RPY) angles during the trajectory tracking experiment. The RPY angles provide additional insight into the orientation of the UAV throughout the test, complementing the position and velocity data. Analysis of these angles allows for a better understanding of the vehicle’s attitude control performance and its ability to maintain stable orientation while following the desired trajectory. Figure 7a and b show the variation in thrust and control torques, respectively, generated by the three functioning rotors during the figure-eight maneuver. The presented results, encompassing trajectory profiles, tracking errors, attitude angles, and angular velocities, consistently demonstrate the quadrotor’s ability to stabilize its orientation and successfully track the desired trajectory, even in the event of a complete failure of one actuator.

#### Robustness analysis under parametric uncertainty without disturbances

To assess the robustness of the proposed control system to parameter variations, a Monte Carlo analysis was performed with respect to the quadrotor parameters eg. mass. The nominal mass was set to $m = 1$ kg, and its value was randomly sampled from a uniform distribution within $\pm 50\%$ of the nominal value, resulting in $N_{\text {trials}} = 30$ simulation runs with different mass realizations. For each trial, the closed-loop tracking error was recorded over time for all position axes (*x*, *y*, *z*).

**Fig. 8:**
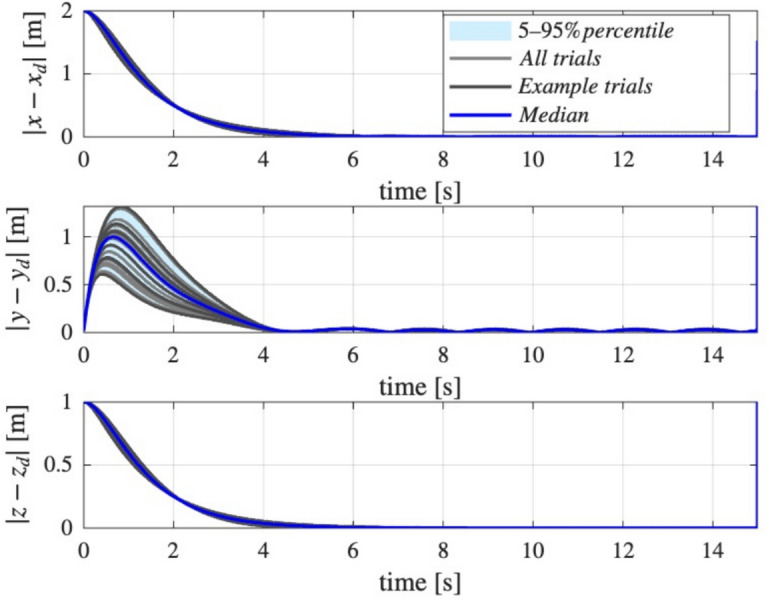
Monte Carlo envelope of position tracking errors under $50\%$ mass uncertainty: robustness analysis for the trajectory following task without external disturbances.

To visualize the variability of the tracking performance under mass uncertainty, an envelope plot was generated for each axis (Fig. 8). The plot shows the median and 5th–95th percentile range of the absolute position tracking errors across all trials at each time instant, as well as individual sample trajectories. This approach provides a comprehensive view of both typical and worst-case controller performance under significant parametric uncertainty. The results demonstrate the ability of the proposed control method to maintain satisfactory tracking accuracy, despite large deviations in system mass.

### Trajectory tracking performance under external disturbances

#### Disturbance models

- Sinusoidal disturbance

To provide a baseline robustness test, a simple sinusoidal disturbance was first introduced in the simulation model. The disturbance was applied additively to the translational dynamics of the quadrotor as a time-varying force vector:

65 $$\begin{aligned} \textbf{d}(t) = \begin{bmatrix} A_1 \sin (2\pi t) \\ A_2 \sin (2\pi t) \\ A_3 \sin (2\pi t) \end{bmatrix} \; [\textrm{N}], \end{aligned}$$

where the components correspond to the *x*, *y*, and *z* axes, respectively, and *t* is the simulation time in seconds. Such sinusoidal disturbances acting on the translational dynamics of quadrotors, i.e., as external forces in the *x*, *y*, and *z* directions, are widely used in the literature to evaluate controller robustness ^49^. It should be noted that the nominal mass of the simulated quadrotor is $m = 1$ kg, which corresponds to a gravitational force of $mg \approx 9.8$ N, while the maximum available thrust in the model is 20 N per rotor. Under these conditions, an external disturbance reaching up to 3 N in the *x*, *y*, or *z* directions constitutes a substantial challenge for the control system. Such a disturbance corresponds to approximately $30\%$ of the vehicle weight and a significant fraction of the actuator capability, particularly in the fault–tolerant scenario where only three rotors are operational. This choice was intentional, aiming to demonstrate the controller’s ability to maintain performance and trajectory tracking under severe and rapidly varying perturbations.

(b) Stochastic turbulence and gusts

To demonstrate robustness more convincingly, a stochastic disturbance model was adopted. The external forces were modeled as a superposition of three components commonly used to approximate atmospheric turbulence and wind gusts ^50–52^: a high–frequency Ornstein–Uhlenbeck (OU) process $\textbf{f}_{\textrm{OU}}(t)$ capturing rapid turbulent fluctuations, a low–frequency OU process $\textbf{f}_{\textrm{LF}}(t)$ representing slow drift and quasi–steady wind biases, and a Poisson shot–noise process $\textbf{f}_{\textrm{gust}}(t)$ modeling impulsive gusts with exponential decay. An additional aerodynamic drag term $\textbf{f}_{\textrm{drag}}(t)=-C\,\textbf{v}(t)$ with $C=\textrm{diag}(0.10,\,0.10,\,0.20)\,\mathrm {N\cdot s/m}$ was included to account for baseline aerodynamic resistance. The total disturbance force is defined as:

$$\textbf{f}_{\textrm{dist}}(t) = \textbf{f}_{\textrm{OU}}(t) + \textbf{f}_{\textrm{LF}}(t) + \textbf{f}_{\textrm{gust}}(t) + \textbf{f}_{\textrm{drag}}(t).$$

For the multirotor UAV considered in this study ($m=1\,\textrm{kg}$), the parameters were selected such that the root–mean–square (RMS) disturbance level corresponds to approximately 5–$10\%$ of the vehicle weight ($mg \approx 9.8\,\textrm{N}$), consistent with typical small–scale quadrotor tests. The adopted values were^53^: high–frequency turbulence $\tau _{\textrm{OU}} = [0.18,\,0.18,\,0.24]\,\textrm{s}$, $\sigma _{\textrm{OU}} = [0.55,\,0.55,\,0.80]\,\textrm{N}$; low–frequency drift $\tau _{\textrm{LF}} = [7,\,7,\,9]\,\textrm{s}$, $\sigma _{\textrm{LF}} = [0.24,\,0.24,\,0.35]\,\textrm{N}$; and wind gusts $\tau _{\textrm{gust}} = [0.30,\,0.30,\,0.45]\,\textrm{s}$, $\lambda _{gust} = 0.45\,\textrm{s}^{-1}$, $A_{\textrm{gust}}^{\textrm{rms}} = [0.75,\,0.75,\,1.05]\,\textrm{N}$. These settings represent a non-negligible disturbance level for a small, slowly flying quadrotor platform. The chosen model is in line with standard stochastic turbulence representations (e.g., Dryden or Lüss models) commonly used in UAV robustness studies.

**Fig. 9:**
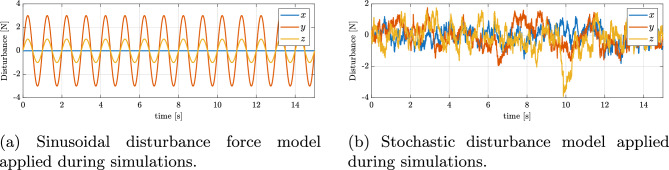
Disturbance models considered in this study: (**a**) sinusoidal disturbance model, (**b**) stochastic turbulence and gust disturbance model.

The two disturbance profiles along with their time histories are presented in Fig. 9, ensuring transparency and reproducibility of the simulation scenarios.

#### Results for trajectory following with external disturbances

**Fig. 10:**
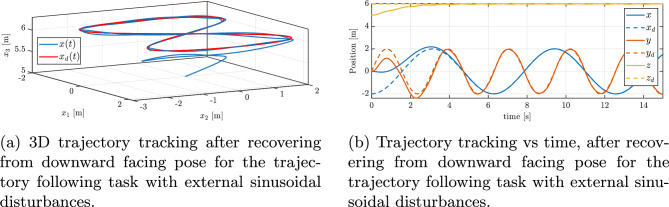
Trajectory tracking performance with external sinusoidal disturbances: (**a**) 3D trajectory, (**b**) position vs. time.

**Fig. 11:**
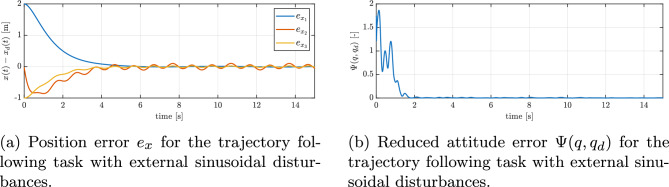
Tracking errors under external sinusoidal disturbances: (**a**) position error, (**b**) reduced attitude error.

Figure 10a and b illustrate the quadrotor’s adherence to a ‘figure-of-8’ reference path following with disturbances. Initially, a notable attitude error results in a temporary divergence from the set path, as is evident from the position errors graph in Fig. 11a. The controller rapidly corrects the orientation, and the quadrotor converges to the prescribed path. Once this recovery is complete, the UAV closely follows the reference curve. Despite the disturbances, the trajectory is tracked correctly and the tracking error values are kept within acceptable bounds by the control system.

**Fig. 12:**
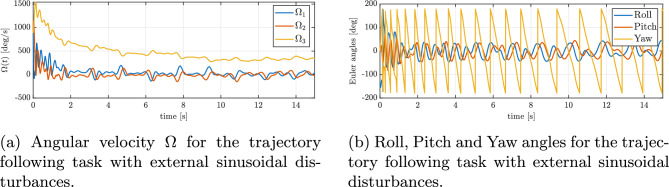
Attitude dynamics under external sinusoidal disturbances: (**a**) angular velocities, (**b**) Euler angles.

**Fig. 13:**
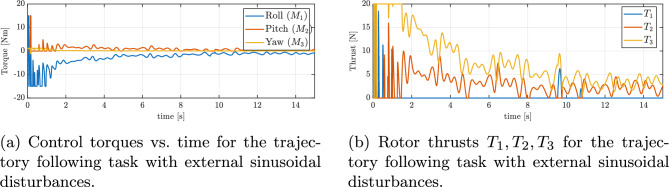
Control inputs under external sinusoidal disturbances: (**a**) commanded torques, (**b**) rotor thrusts.

In Fig. 11b, the progression of the minimized attitude error function $\Psi \left( q, q_{d}\right)$ throughout the maneuver is depicted, demonstrating an exponential decline of $\Psi$ towards a negligibly small oscilations around zero in the case of disturbances. This confirms that the controller swiftly reduces the orientation mismatch caused by the disturbances and the UAV’s orientation is brought back under control almost as quickly as it would be without disturbances. Furthermore, Figure 12a and b present the angular velocities and Euler angles respectively, around the three principal axes of the body during the maneuver, highlighting a swift escalation and subsequent saturation of the angular velocity around the body’s *z* axis, attributed to rotational drag. Despite strong disturbances, these parameters stabilize and are maintained at an appropriate level. Figure 13a depicts the control torques over time. There is an evident peak in the control torque commands at the start of the maneuver–this is when the controller is exerting maximum effort to recover from the large attitude error and counteract the immediate effect of the disturbances. These initial high torque demands show that the controller utilizes the available control authority aggressively but in a controlled manner. After the vehicle’s attitude is corrected and the trajectory is rejoined, the required control torques drop to lower magnitudes. They continue to fluctuate in response to the ongoing sinusoidal disturbances, but only within a moderate range. The absence of any runaway increase in control effort and the lack of long-term saturation in Fig. 13a indicate that the controller is coping well with the disturbances and one motor loss.

**Fig. 14:**
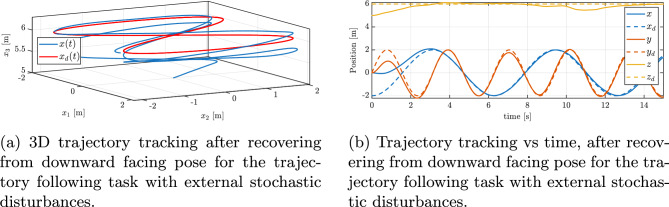
Trajectory tracking performance with external stochastic disturbances: (**a**) 3D trajectory, (**b**) position vs. time.

**Fig. 15:**
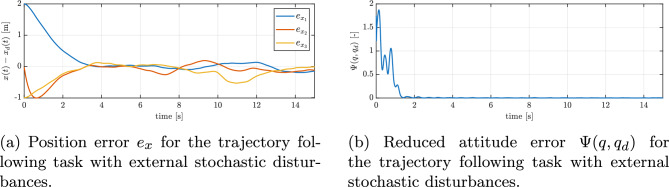
Tracking errors under external stochastic disturbances: (**a**) position error, (**b**) reduced attitude error.

**Fig. 16:**
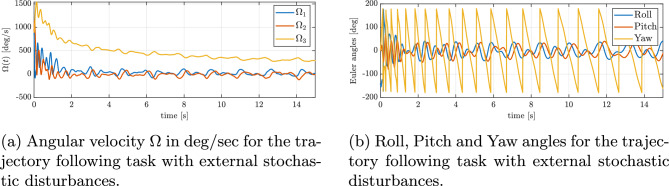
Attitude dynamics under external stochastic disturbances: (**a**) angular velocities, (**b**) Euler angles.

**Fig. 17:**
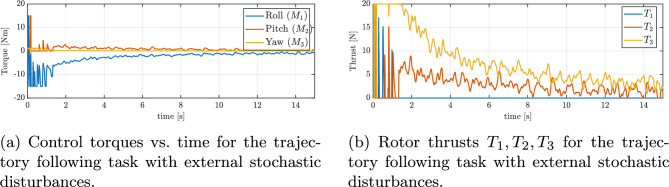
Control inputs under external disturbances: (**a**) commanded torques, (**b**) rotor thrusts.

Figure 13b illustrates the changes in thrust produced by the three operational rotors throughout the maneuver in the case with disturbances. In contrast to a disturbance-free scenario, here the thrusts vary frequently and rapidly. This high-frequency modulation of thrust corresponds directly to the controller counteracting the external forces: whenever a disturbance force pushes the vehicle off course, the controller immediately adjusts rotor thrusts to compensate. One can see small oscillatory patterns in the thrust plots, which match the frequency of the injected sinusoidal disturbances (as described in Section “Disturbance models”). Crucially, throughout the maneuver, all thrust values remain within the allowed range (none of the rotors hit the 20N saturation limit for an extended period). The ability to modulate thrust quickly and effectively, without saturating, demonstrates the system’s capacity to reject disturbances while still respecting actuator limits. As a result, the quadrotor achieves stable flight and trajectory tracking despite the continuous disturbances. Figure 14a and b illustrate the quadrotor’s performance in tracking the same reference trajectory (figure-of-8’) under stochastic turbulence and gust disturbances. Compared with the sinusoidal case, the presence of random fluctuations results in temporary larger deviations from the desired path, as seen in the position error plot in Fig. 15a. Despite these transient excursions, the control system consistently drives the vehicle back toward the reference trajectory, and the UAV successfully maintains bounded tracking errors throughout the maneuver. In Fig. 15b, the evolution of the minimized attitude error function $\Psi (q,q_d)$ is shown. Although the disturbance injection produces irregular fluctuations, $\Psi$ still exhibits a clear exponential decline toward small values, with only minor stochastic oscillations around zero. This behavior confirms that the controller remains capable of rapidly mitigating orientation mismatches even under random gust-like perturbations. Furthermore, Fig. 16a and b present the angular velocities and Euler angles, respectively, during the maneuver. Short-lived spikes in angular rates are visible, especially around the body *z* axis, caused by impulsive gust events. Nevertheless, these quantities quickly stabilize, and no long-term drift is observed in roll or pitch angles. The yaw dynamics remain bounded in their fluctuations, showing that the stochastic inputs do not destabilize the system. Finally, Fig. 17a and b depicts the commanded control torques and rotor thrusts respectively. Similar to the sinusoidal disturbance case, there is an initial demand peak associated with large initial attitude correction. During the subsequent trajectory tracking, the torque signals exhibit irregular variations in response to the stochastic disturbance. Importantly, these variations remain moderate, with no evidence of control saturation or loss of authority. Overall, the results demonstrate that the proposed geometric controller effectively preserves trajectory tracking performance even under stochastic turbulence and gust disturbances, with only short-lived deviations that are rapidly corrected.

### Single motor failure in a hover scenario

This simulation was designed explicitly to replicate the hover experiment conducted on the real quadrotor. We have prepared the same scenario as in the laboratory test - the vehicle takes off and hovers on desired altitude. At t=10s, one motor is permanently disabled to emulate failure. The reported outputs match the experimental readouts - attitude angles (roll, pitch, yaw), body angular rates, altitude. The multirotor mathematical model, actuator mapping, and inertial parameters are identical to those used in the previous sections, so that the numerical results are directly comparable regarding measured signals (angular velocities and Euler angles). The mass and moments of inertia of the quadrotor were unchanged from the earlier simulations. Following the laboratory conditions, we do not inject exogenous disturbances (e.g., wind gusts, sinusoidal). Up to t=10s, the baseline PID loops regulate position and attitude during take of and hovering. At t=10s, motor 4 is set to zero thrust and the control law is switched to the geometric controller for attitude/position regulation under the reduced actuation. In order to consistently simulate, and later reproduce in experiments, the transition from quadcopter operation to a tricopter configuration after one full rotor loss, it is necessary to define an appropriate control allocation matrix. This matrix ensures that the remaining actuators are correctly mapped to the reduced set of control inputs, enabling seamless switching between the two configurations. For a quadrotor in the “*X*” configuration, the control allocation matrix that maps individual rotor thrusts $T_i$ to the total thrust and torques is given by:

$$\begin{bmatrix} f \\ M_1 \\ M_2 \\ M_3 \end{bmatrix} = \begin{bmatrix} b & b & b & b \\ b l \tfrac{1}{\sqrt{2}} & b l \tfrac{1}{\sqrt{2}} & -b l \tfrac{1}{\sqrt{2}} & -b l \tfrac{1}{\sqrt{2}} \\ - b l \tfrac{1}{\sqrt{2}} & b l \tfrac{1}{\sqrt{2}} & b l \tfrac{1}{\sqrt{2}} & - b l \tfrac{1}{\sqrt{2}} \\ - d & d & d & - d \end{bmatrix} \begin{bmatrix} T_1 \\ T_2 \\ T_3 \\ T_4 \end{bmatrix}$$

In the case of a rotor failure (e.g., rotor 4 is inoperative), the system becomes a tricopter. The reduced allocation matrix becomes:

$$\begin{bmatrix} f \\ M_1 \\ M_2 \end{bmatrix} = \begin{bmatrix} b & b & b \\ b l \tfrac{1}{\sqrt{2}} & b l \tfrac{1}{\sqrt{2}} & -b l \tfrac{1}{\sqrt{2}} \\ - b l \tfrac{1}{\sqrt{2}} & b l \tfrac{1}{\sqrt{2}} & b l \tfrac{1}{\sqrt{2}} \\ \end{bmatrix} \begin{bmatrix} T_1 \\ T_2 \\ T_3 \end{bmatrix}$$

**Fig. 18:**
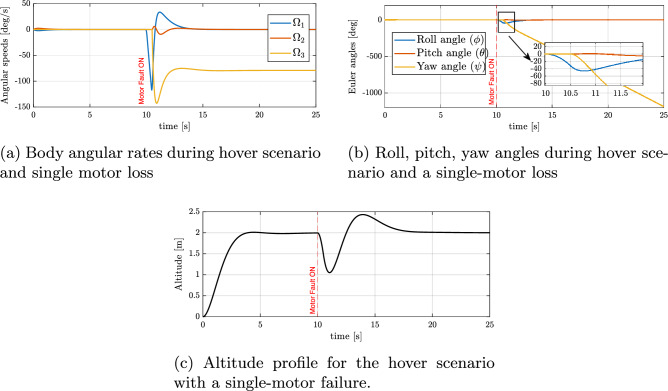
Quadrotor hover scenario used to assess the geometric controller under a single-motor failure. (**a**) Body angular rates, (**b**) Euler angles, (**c**) Altitude profile.

The conducted simulations and presented figure set reproduce the future experimental setup and measurements: (a) RPY angles (Fig. 18b) with the failure time marked by a vertical dashed line, (b) body rates (Fig. 18a), and (c) altitude (Fig. 18c). At $t=10$ s, the motor failure becomes evident as a distinct disturbance across all signals. Nevertheless, the vehicle quickly compensates for the fault, and within approximately 7 s it successfully returns to the desired hover altitude and operating point, confirming the effectiveness of the proposed control allocation and geometric strategy. As expected, the yaw angle exhibits unbounded growth since the loss of a single rotor eliminates control authority about the yaw axis; consequently, the yaw rate also does not converge back to zero. This behavior is fully consistent with previous findings reported in the literature, where aggressive trajectory tracking or hover recovery under rotor loss leads to inevitable yaw drift while maintaining stable reduced–attitude and altitude tracking.

### Robustness analysis under parametric uncertainty with disturbances

To further evaluate the robustness of the proposed control strategy, an additional Monte Carlo analysis was conducted for the scenario involving both parametric uncertainties and external disturbances. Similar to the analysis without disturbances, the quadrotor mass was again set nominally to $m = 1$ kg and varied randomly within a $\pm 50\%$ range using a uniform distribution. For each of the $N_{\text {trials}} = 30$ simulation runs, distinct mass values were selected, and the system was subjected to predefined external disturbance signals during trajectory tracking.

**Fig. 19:**
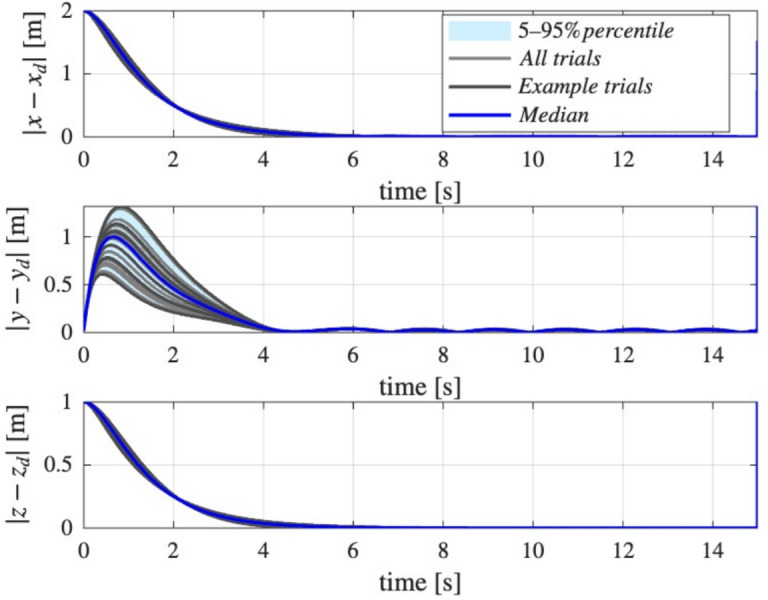
Monte Carlo envelope of position tracking errors under $50\%$ mass uncertainty: robustness analysis for the trajectory following task with external sinusoidal disturbances.

For every trial, the closed-loop tracking errors in the *x*, *y*, and *z* axes were recorded over the simulation horizon. The results were summarized using envelope plots (see Fig. 19), which depict the median and the 5th–95th percentile range of the absolute position tracking errors across all trials at each time instant. Representative sample trajectories are also included to illustrate the spread of possible outcomes.

This comprehensive visualization reveals both the typical and worst-case performance of the controller when confronted simultaneously with significant parameter uncertainty and external disturbances. The observed results confirm that the proposed control system can achieve robust tracking performance, maintaining satisfactory accuracy even under challenging real-world conditions that combine mass variation and disturbance inputs.

## Preliminary experimental validation

**Fig. 20:**
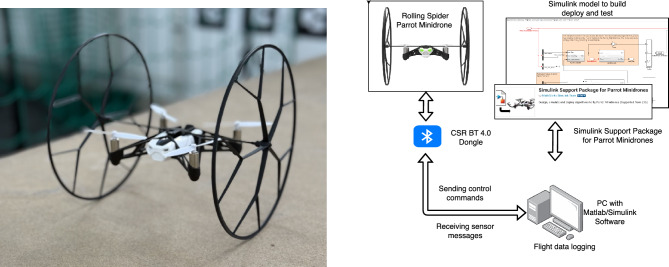
Experimental platform used for evaluation: (left) the Parrot Rolling Spider quadrotor and (right) the schematic representation of the experimental system.

This section provides an end-to-end hardware validation of the proposed nonlinear geometric control framework in the representative hover scenario. The complete controller is executed in real time. The goal is to validate the full approach (control law, and actuation/allocation) under safety-constrained conditions. The rotor-failure condition was enforced to match the simulation fault model. Due to platform and facility constraints, the physical tests were restricted to hover-level maneuvers after rotor loss. More aggressive recoveries (e.g., inverted flips) are evaluated in simulation and are not attempted on the current testbed. Experimental tests were conducted on a Parrot Rolling Spider quadrotor (Fig. 20). The manufacturer’s SDK and communication protocol allowed full access to the onboard control loops, enabling implementation and real-time testing of the developed algorithms via MATLAB/Simulink and ROS environments. In this case, to implement the proposed geometric controller on a real quadrotor platform, we utilized the MATLAB/Simulink Support Packages for Parrot drone hardware. This toolchain enables seamless integration between model-based design and hardware deployment. Specifically, the controller algorithms were developed and tested within the Simulink environment, and the corresponding C code was automatically generated using Matlab/Simulink Coder. The generated code was then deployed directly to the Rolling Spider drone hardware, enabling real-time execution and experimental validation of the control strategies in real-world conditions. This approach significantly streamlines the transition from simulation to initial practical implementation, facilitating rapid prototyping and iterative refinement of control algorithms.

It is important to note that the Parrot Rolling Spider platform provides only a limited total thrust, which is insufficient to maintain hovering flight with only three operating rotors. Consequently, while attitude (roll and pitch) control can be preserved under fault conditions, the vehicle is unable to sustain altitude and will gradually descend. Nevertheless, this experimental setup still allows us to verify whether the geometric controller is able to maintain the drone’s orientation even under conditions that are more demanding than scenarios with sufficient total thrust, thus providing a particularly stringent test of the control algorithm’s effectiveness.

During the experiments, a complete failure of one rotor was emulated by intentionally switching off one motor in flight (at 7s, during hovering). Two scenarios were investigated: (i) with the baseline controller (standard one without geometric approach), and (ii) with the proposed geometric nonlinear controller). For both cases, the time evolution of the roll, pitch, and yaw angles (RPY), as well as angular rates (pqr), were recorded, providing quantitative insight into the system’s behavior during and after the motor fault.

**Fig. 21:**
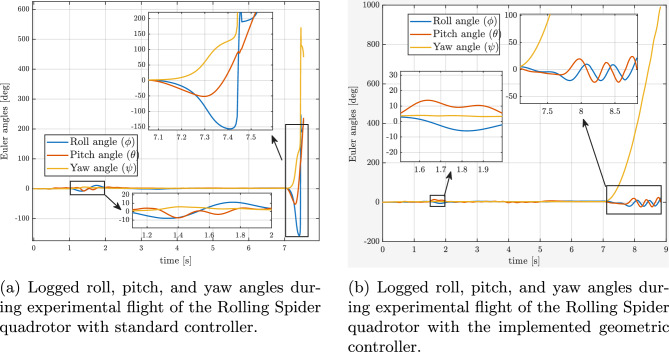
Experimental attitude profiles (roll, pitch, yaw) of the Rolling Spider quadrotor: (**a**) with standard controller, (**b**) with geometric controller.

**Fig. 22:**
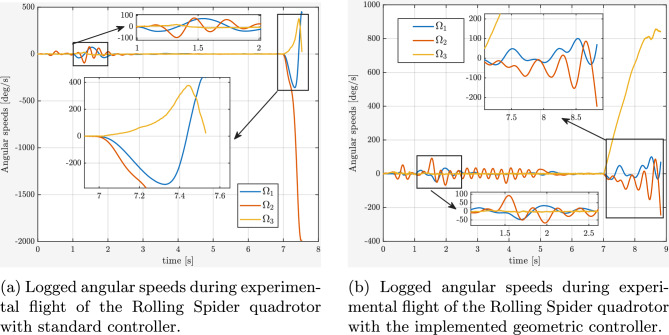
Experimental angular speed profiles of the Rolling Spider quadrotor: (**a**) with standard controller, (**b**) with geometric controller.

**Fig. 23:**
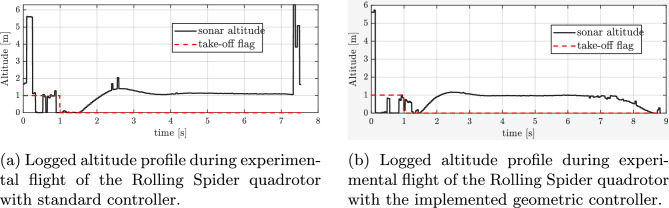
Experimental altitude profiles of the Rolling Spider quadrotor: (**a**) with standard controller, (**b**) with geometric controller.

The results from these tests (Figs. 21a, 22 and 23b) demonstrate a clear contrast between the two scenarios. Without the proposed geometric controller, the UAV is unable to compensate for the asymmetric loss of thrust, resulting in rapid divergence of the attitude angles and angular velocities–particularly along the axis corresponding to the failed rotor. Roll and pitch angles are not stabilized. This leads to a loss of control authority, resulting in a rapid rollover and an uncontrolled flip that ends in a crash. The roll and pitch angles exhibit unbounded growth, as visualized in Fig. 21a, while Fig. 22a shows the dramatic increase of angular rates in the affected axis. In the absence of geometric control, the reading from the ultrasonic altitude sensor (Fig. 23a at the moment of motor failure and the sudden flip of the quadrotor shows an abrupt spike in the measured altitude instead of a decrease. This effect is caused by the fact that, immediately after the failure, the drone is instantaneously and uncontrollably inverted, which leads to incorrect altitude measurements This effect is caused by the fact that, immediately after the failure, the drone is instantaneously and uncontrollably inverted, which leads to incorrect altitude measurements (sonar’s orientation towards the top, not the ground).

In contrast, when the geometric algorithm is enabled, the UAV is able to maintain stable control of the roll and pitch angles within a safe range despite the loss of one actuator. Although the quadrotor cannot maintain its altitude - an inherent limitation under complete rotor loss - it executes a controlled descent and achieves a soft landing (see Fig. 23b). The attitude (Fig. 21b) and angular velocity (Fig. 22b) profiles confirm that the system remains stable, with no sign of overturning or uncontrolled spinning. This soft and coordinated descent is qualitatively superior to the uncontrolled crash observed without geometric controller.

These experimental findings provide some initial evidence for the effectiveness of the proposed geometric control scheme under real-world actuator faults. By reporting both RPY and pqr profiles in both scenarios, the experiments go well beyond pure simulation and demonstrate the practical applicability and potential of the developed control methodology (https://youtu.be/0GdCECVQnew).

## Concluding remarks

In this work, we proposed a novel fault-tolerant geometric control law for a quadrotor UAV experiencing a complete failure of a single rotor. The presented design effectively exploits the underlying geometric structure of the reduced configuration manifold, allowing the UAV to retain controllability over its position and reduced attitude despite the significant loss of actuation authority. In contrast to existing fault-tolerant approaches, the proposed method enables the execution of aggressive maneuvers, including attitude recovery from inverted configurations and nontrivial trajectory tracking, without being restricted by singularities or degraded performance envelopes. These aggressive scenarios are demonstrated in simulation (including sinusoidal disturbances and stochastic gusts stress tests), whereas the hardware validation targets a one motor failed scenario during hover.

A key contribution lies in the development of a backstepping-based geometric controller for reduced attitude tracking on the Lie group SO(3), which is inherently free from singularities and capable of supporting arbitrarily high angular rates. This capability was essential to suppress large transient responses and maintain high-fidelity tracking, particularly during aggressive recovery and maneuvering scenarios. Furthermore, by extending the control design to SE(3) with a bounded, saturation-based position feedback law, the system achieves exponential convergence of the full-state tracking errors for a broad set of initial conditions, providing robust performance guarantees in the presence of the underactuation introduced by rotor loss.

Practical considerations related to implementation were also discussed. Specifically, it was observed that for a conventional quadrotor platform, where each rotor can only generate positive thrust, achieving sufficient angular momentum about the body-fixed vertical axis is critical when initiating reduced attitude and position tracking maneuvers after fault detection. Consequently, the control law incorporates a strategy to first increase the angular rate around the body z-axis to enable stable and effective recovery and trajectory tracking under fault conditions.

In addition to nominal trajectory tracking, further simulations were conducted to assess the robustness of the proposed controller under external disturbances. Both sinusoidal perturbations and stochastic turbulence–gust models were introduced, representing structured and realistic atmospheric effects, respectively. In both cases, the control system successfully maintained the quadrotor close to the desired trajectory, suppressing oscillations and compensating for transient deviations. These results confirm that the controller can handle both deterministic and random disturbance profiles without significant degradation in tracking accuracy. Moreover, a simulation scenario identical to the preliminary experimental setup was considered, where a single rotor was disabled at hover. The results demonstrated that, provided the platform retains sufficient thrust margin, the proposed controller is able to stabilize the vehicle, regulate its altitude, and preserve reduced attitude tracking, despite the unavoidable yaw drift caused by loss of control authority around the vertical axis. This observation is fully aligned with experimental findings and prior theoretical analyses, reinforcing the practical viability of the approach in safety–critical conditions.

Numerical simulation and preliminary experimental results demonstrate the potential of the proposed control strategy to maintain performance in the presence of severe actuator faults. Future work will focus on extending the proposed framework to handle multiple rotor failures, incorporating real-time fault detection and isolation (FDI) mechanisms, and validating the approach through extensive trajectory tracking experiments on physical quadrotor platform with sufficient thrust margin to enable stable flight after complete rotor loss. The results presented herein offer a significant step towards resilient UAV operation in safety-critical applications where actuator redundancy cannot be assumed.

## Future work

Future research will extend the proposed fault-tolerant geometric control framework with intelligent methods to enhance robustness, adaptability, and autonomy under uncertainties and actuator constraints. Promising directions include hybrid strategies combining sliding mode and predictive nonlinear approaches for mitigating unknown dynamics and external disturbances in severe rotor loss scenarios. Logic-based switching schemes ^54^ and event-triggered updates may further improve transient performance and stability during abrupt actuator failures. Data-driven and learning-based methods, such as neural network adaptation ^55^, will also be explored to improve online disturbance estimation and enable cooperative fault-tolerant control in multi-agent UAV systems. Ongoing experiments with a modified Crazyflie 2.1 platform aim to validate the approach under rotor failures and assess practical issues such as sensor noise, delays, and embedded hardware limitations. Together, these efforts seek to bridge geometric control theory with robust switching and learning-based methods, moving toward resilient UAV operation in uncertain and safety-critical environments.

## Data Availability

The data has been generated during the study and will be available on reasonable request to the corresponding author.

## Appendix 1 - Nomenclature

**Table:**

Symbol	Description
*x*	Position vector of the quadrotor’s in the inertial frame ($\mathbb {R}^3$)
*v*	Velocity of the center of mass in the inertial frame ($\mathbb {R}^3$)
*R*	Rotation matrix (attitude) from body frame to inertial frame (*SO*(3))
${\Omega }$	Angular velocity vector in body frame ($\mathbb {R}^3$)
*J*	Inertia matrix of the quadrotor in body frame ($3 \times 3$)
$J_r$	Rotor inertia ($\text {kg}\,\text {m}^2$)
*m*	Mass of the quadrotor ($\text {kg}$)
$T_i$	Thrust produced by the *i*-th rotor ($\text {N}$)
${D}_i$	Drag torque produced by the *i*-th rotor ($\text {Nm}$)
*f*	Total thrust generated by all operating rotors ($\text {N}$)
*M*	Control torque vector ($\text {Nm}$)
$M_1, M_2, M_3$	Control torques about body axes ($\text {Nm}$)
*l*	Distance from the quadrotor’s center to each rotor ($\text {m}$)
$C_l$	Aerodynamic lift coefficient
$C_d$	Aerodynamic drag coefficient
*g*	Gravitational acceleration ($9.81\,\text {m}/\text {s}^2$)
${e}_1, {e}_2, {e}_3$	Standard basis vectors of inertial frame
${b}_1, {b}_2, {b}_3$	Standard basis vectors of body frame
*q*	Reduced attitude (thrust axis direction), ${q} = {Re}_3$ (${S}^2$)
${q}_d$	Desired reduced attitude trajectory (${S}^2$)
${x}_d$	Desired position trajectory ($\mathbb {R}^3$)
${e}_x$	Position tracking error (${x} - {x}_d$)
${e}_v$	Velocity tracking error (${v} - \dot{x}_d$)
*U*	Control torque vector (input) $[U_1, U_2]^T$
$U_1, U_2$	Control torques for reduced attitude (roll/pitch)
${\tau }_d$	Aerodynamic rotational drag torque ($\text {Nm}$)
$\Psi ({q}, {q}_d)$	Reduced attitude error function
${e}_q$	Reduced attitude error vector
${e}_{\Omega }$	Angular velocity error vector
$\Delta$	Bound for disturbances/uncertainties in the model
$k_q, k_{\Omega }$	Controller gains (reduced attitude, angular rate)
$k_x, k_v$	Position and velocity controller gains
$\sigma _1, \sigma _2$	Smooth saturation functions for position controller
${f}_d$	Feedforward term for position tracking
$A_1, A_2, A_3$	Amplitudes of external disturbances in *x*, *y*, *z* directions

## References

[1] Beard, R. Quadrotor dynamics and control Rev 0.1. BYU ScholarsArchive (1), 1325 (2008).

[2] Lupashin, S., Schöllig, A., Sherback, M. & D’Andrea, R. A simple learning strategy for high-speed quadrocopter multi-flips. In *2010 IEEE International Conference on Robotics and Automation (ICRA)* 1642–1648 (IEEE, 2010).

[3] Hehn, M. & D’Andrea, R. A flying inverted pendulum. In *2011 IEEE International Conference on Robotics and Automation (ICRA)* 763–770 (IEEE, 2011).

[4] Müller, M., Lupashin, S. & D’Andrea, R. Quadrocopter ball juggling. In *2011 IEEE/RSJ International Conference on Intelligent Robots and Systems (IROS)* 5113–5120 (IEEE, 2011).

[5] Mellinger, D. & Kumar, V. Minimum snap trajectory generation and control for quadrotors. In *2011 IEEE International Conference on Robotics and Automation (ICRA)* 2520–2525 (IEEE, 2011).

[6] Thomas, J., Polin, J., Sreenath, K. & Kumar, V. Avian-inspired grasping for quadrotor micro UAVs. In *ASME 2013 International Design Engineering Technical Conferences and Computers and Information in Engineering Conference, 2013* V06AT07A014 (American Society of Mechanical Engineers, 2013).

[7] Cutler, M. J. Design and control of an autonomous variable-pitch quadrotor helicopter (2012).

[8] Cutler, M. & How, J. P. Actuator constrained trajectory generation and control for variable-pitch quadrotors. In *AIAA Guidance, Navigation, and Control Conference, 2012* 1–15 (2012).

[9] Cutler, M. & How, J. P. Analysis and control of a variable-pitch quadrotor for agile flight. *J. Dyn. Syst. Meas. Control Trans. ASME* **137**(10), 101002. 10.1115/1.4030676 (2015).

[10] Sheng, S. & Sun, C. Control and optimization of a variable-pitch quadrotor with minimum power consumption. *Energies* **9**(4), 232 (2016).

[11] Gupta, N., Kothari, M. et al.: Flight dynamics and nonlinear control design for variable-pitch quadrotors. In *American Control Conference (ACC), 2016* 3150–3155 (American Automatic Control Council (AACC), 2016).

[12] Ambroziak, L., Ołdziej, D. & Koszewnik, A. Multirotor motor failure detection with piezo sensor. *Sensors* **23**(2), 1048. 10.3390/s23021048 (2023). 36679844 10.3390/s23021048 PMC9862776

[13] Wang, X., Sun, S., Kampen, E.-J. & Chu, Q. Quadrotor fault tolerant incremental sliding mode control driven by sliding mode disturbance observers. *Aerosp. Sci. Technol.* **87**, 417–430 (2019).

[14] Nguyen, N. P. & Hong, S. K. Fault-tolerant control of quadcopter UAVs using robust adaptive sliding mode approach. *Energies* **12**(1), 95. 10.3390/en12010095 (2019).

[15] Besnard, L., Shtessel, Y. B. & Landrum, B. Quadrotor vehicle control via sliding mode controller driven by sliding mode disturbance observer. *J. Frankl. Inst.* **349**(2), 658–684 (2012).

[16] Ambroziak, L., Simha, A., Pawluszewicz, E., Kotta, U., Bożko, A. & Kondratiuk, M. Motor failure tolerant control system with self diagnostics for unmanned multirotors. In *2019 24th International Conference on Methods and Models in Automation and Robotics (MMAR), Miedzyzdroje, Poland* 422–427. 10.1109/MMAR.2019.8864726 (2019).

[17] Xu, D., Whidborne, J. F. & Cooke, A. Fault tolerant control of a quadrotor using C_1_ adaptive control. *Int. J. Intell. Unmanned Syst.* **4**(1), 43–66 (2016).

[18] Goodarzi, F. A., Lee, D. & Lee, T. Geometric adaptive tracking control of a quadrotor unmanned aerial vehicle on SE(3) for agile maneuvers. *J. Dyn. Syst. Meas. Control* **137**(9), 91007 (2015).

[19] Guo, Y., Jiang, B. & Zhang, Y. A novel robust attitude control for quadrotor aircraft subject to actuator faults and wind gusts. *IEEE CAA J. Autom. Sin.* **5**(1), 292–300 (2018).

[20] Nan, F., Sun, S., Foehn, P. & Scaramuzza, D. Nonlinear MPC for quadrotor fault-tolerant control. *IEEE Robot. Autom. Lett.* **7**(2), 5047–5054. 10.1109/LRA.2022.3154033 (2022).

[21] Sun, S., Cioffi, G., Visser, C. & Scaramuzza, D. Autonomous quadrotor flight despite rotor failure with onboard vision sensors: Frames vs events. *IEEE Robot. Autom. Lett.* **6**(2), 580–587. 10.1109/LRA.2020.3048875 (2021).

[22] Yu, X., Zhou, S., Guo, K., Zhang, Y. & Guo, L. Integrated reconfiguration mechanism for quadrotors with capability analysis against rotor failure. *J. Guidance Control Dyn.* (2022)

[23] Mueller, M. W. & D’Andrea, R. Stability and control of a quadrocopter despite the complete loss of one, two, or three propellers. In *2014 IEEE International Conference on Robotics and Automation (ICRA)* 45–52 (IEEE, 2014)

[24] Mueller, M. W. & D’Andrea, R. Relaxed hover solutions for multicopters. *Int. J. Robot. Res.* **35**(8), 873–889. 10.1177/0278364915596233 (2016).

[25] Lippiello, V., Ruggiero, F. & Serra, D. Emergency landing for a quadrotor in case of a propeller failure: A backstepping approach. In *2014 IEEE/RSJ International Conference on Intelligent Robots and Systems* 4782–4788 (IEEE, 2014)

[26] Lippiello, V., Ruggiero, F. & Serra, D. Emergency landing for a quadrotor in case of a propeller failure: A PID based approach. In *12th IEEE International Symposium on Safety, Security and Rescue Robotics, SSRR 2014 - Symposium Proceedings*. 10.1109/SSRR.2014.7017647 (2014).

[27] Freddi, A., Lanzon, A. & Longhi, S. A feedback linearization approach to fault tolerance in quadrotor vehicles. *IFAC Proc. Vol.* **44**(1), 5413–5418. 10.3182/20110828-6-IT-1002.02016 (2011).

[28] Lanzon, A., Freddi, A. & Longhi, S. Flight control of a quadrotor vehicle subsequent to a rotor failure. *J. Guidance Control Dyn.* **37**(2), 580–591. 10.2514/1.59869 (2014).

[29] Lu, P. & Kampen, E.-J. Active fault-tolerant control for quadrotors subjected to a complete rotor failure. In *2015 IEEE/RSJ International Conference On Intelligent Robots and Systems (IROS)* 4698–4703 (IEEE, 2015).

[30] Akhtar, A., Waslander, S. L. & Nielsen, C. Fault tolerant path following for a quadrotor. In *52nd IEEE Conference on Decision and Control* 847–852 (IEEE, 2013).

[31] Gu, X. & Tian, Y. Safety control for the quadrotor unmanned aerial vehicle despite complete lose of one rotor. *IEEE Access* **11**, 124624–124633. 10.1109/ACCESS.2023.3330230 (2023).

[32] Maithripala, D. H. S., Berg, J. M. & Dayawansa, W. P. Almost-global tracking of simple mechanical systems on a general class of Lie groups. *IEEE Trans. Autom. Control* **51**(2), 216–225. 10.1109/TAC.2005.862219 (2006).

[33] Bullo, F. *Geometric Control of Mechanical Systems* Vol. 49 (Springer, 2005).

[34] Lee, T., Leok, M., & McClamroch, N. H. Geometric tracking control of a quadrotor UAV on SE(3). In *2010 49th IEEE Conference On Decision and Control (CDC)* 5420–5425 (IEEE, 2010).

[35] Lee, T. Robust adaptive attitude tracking on with an application to a quadrotor UAV. *IEEE Trans. Control Syst. Technol.* **21**(5), 1924–1930 (2013).

[36] Wu, S., Liang, X., Fang, Y. & He, W. Geometric maneuvering for underactuated VTOL vehicles. *IEEE Trans. Autom. Control* **69**(3), 1507–1519. 10.1109/TAC.2023.3324268 (2024).

[37] Bullo, F., Murray, R. M. & Sarti, A. Control on the sphere and reduced attitude stabilization (1995).

[38] Ramp, M., & Papadopoulos, E. Attitude and angular velocity tracking for a rigid body using geometric methods on the two-sphere. In *2015 European Control Conference (ECC)* 3238–3243 (IEEE, 2015).

[39] Mayhew, C. G. & Teel, A. R. Global stabilization of spherical orientation by synergistic hybrid feedback with application to reduced-attitude tracking for rigid bodies. *Automatica* **49**(7), 1945–1957 (2013).

[40] Khodaverdian, M., Najafi, M., Kazemifar, O. & Rahmanian, S. Fault-tolerant model predictive sliding mode control for trajectory replanning of multi-UAV formation flight. *Appl. Math. Comput.* **487**, 129073. 10.1016/j.amc.2024.129073 (2025).

[41] Bouabdallah, S. & Siegwart, R. Full control of a quadrotor. In *IEEE International Conference on Intelligent Robots and Systems* 153–158. 10.1109/IROS.2007.4399042 (2007).

[42] McCormick, B. W. *Aerodynamics, Aeronautics, and Flight Mechanics* Vol. 2 (Wiley, 1995).

[43] Lee, T. Geometric tracking control of the attitude dynamics of a rigid body on SO(3). In *Proceedings of the 2011 American Control Conference (ACC)* 1200–1205 (IEEE, 2011)

[44] Krstic, M., Kanellakopoulos, I. & Kokotovic, P. V. *Nonlinear and Adaptive Control Design* (Wiley, 1995).

[45] Khalil, H. K. *Noninear Systems* (Prentice-Hall, 1996).

[46] Stein, E. M. & Shakarchi, R. *Real Analysis: Measure Theory, Integration, and Hilbert Spaces* (Princeton University Press, 2009).

[47] Teel, A. R. Global stabilization and restricted tracking for multiple integrators with bounded controls. *Syst. Control Lett.* **18**(3), 165–171. 10.1016/0167-6911(92)90001-9 (1992).

[48] Simha, A., Vadgama, S. & Raha, S. Almost-global exponential tracking of a variable pitch quadrotor on SE(3). *IFAC PapersOnLine* **50**(1), 10268–10273 (2017).

[49] Chen, L., Liu, Z., Dang, Q., Zhao, W. & Wang, G. Robust trajectory tracking control for a quadrotor using recursive sliding mode control and nonlinear extended state observer. *Aerosp. Sci. Technol.* **128**, 107749. 10.1016/J.AST.2022.107749 (2022).

[50] Sadi, M. A., Jamali, A. & Abang Kamaruddin, A. M. N. Optimizing UAV performance in turbulent environments using cascaded model predictive control algorithm and Pixhawk hardware. *J. Braz. Soc. Mech. Sci. Eng.* **47**(8), 396. 10.1007/s40430-025-05693-9 (2025).

[51] Obukhov, S. et al. Modeling wind speed based on fractional Ornstein-Uhlenbeck process. *Energies* **14**(17), 5561. 10.3390/en14175561 (2021).

[52] Pham, K., Nguyen, A. T., Nguyen, L. N. & Van Do Thi, T. Application of the Ornstein-Uhlenbeck process to generate stochastic vertical wind profiles. *J. Aircr.* **62**(4), 1067–1071. 10.2514/1.C038089 (2025).

[53] Abichandani, P., Lobo, D., Ford, G., Bucci, D. & Kam, M. Wind measurement and simulation techniques in multi-rotor small unmanned aerial vehicles. *IEEE Access* **8**, 54910–54927. 10.1109/ACCESS.2020.2977693 (2020).

[54] Fan, D., Zhang, X. & Wen, C. Exponential regulation of uncertain nonlinear triangular impulsive systems: A logic-based switching gain approach. *IEEE Trans. Autom. Control*10.1109/TAC.2025.3545697 (2025).

[55] Li, W., Yue, J., Shi, M., Lin, B. & Qin, K. Neural network-based dynamic target enclosing control for uncertain nonlinear multi-agent systems over signed networks. *Neural Netw.* **184**, 107057. 10.1016/J.NEUNET.2024.107057 (2025). 39721102 10.1016/j.neunet.2024.107057

